# Rhythmic stimulation elicits multiple, concurrent neural responses

**DOI:** 10.1093/cercor/bhag152

**Published:** 2026-09-22

**Authors:** Benjamin J Griffiths, Katharina Duecker, Camille Fakche, Laura Dugué, Andrew Quinn, Ole Jensen

**Affiliations:** Centre for Human Brain Health, University of Birmingham, Birmingham B15 2TT, United Kingdom; School of Psychology, University of Nottingham, Nottingham NG7 2RD, United Kingdom; Department of Neuroscience, Brown University, Providence, Rhode Island 02906, United States; Lyon Neuroscience Research Center, Université de Lyon, Lyon 69500, France; Université Paris Cité, CNRS, Integrative Neuroscience and Cognition Center, Paris 75006, France; Institut Universitaire de France (IUF), Paris, France; Centre for Human Brain Health, University of Birmingham, Birmingham B15 2TT, United Kingdom; Centre for Human Brain Health, University of Birmingham, Birmingham B15 2TT, United Kingdom; Oxford Centre for Human Brain Activity, Department of Psychiatry, University of Oxford, Oxford OX3 7JX, United Kingdom; Department of Experimental Psychology, University of Oxford, Oxford OX1 3EL, United Kingdom

**Keywords:** neural oscillations, rhythmic light stimulation, visual perception, EEG, MEG

## Abstract

Rhythmic light stimulation offers solutions to innumerable cognitive and neurological disorders. However, like any neuromodulatory technique, responses to rhythmic light stimulation are highly variable, producing challenges in replicating lab-based studies and translating findings to the clinic. Across 3 magnetoencephalography (MEG) and electroencephalography (EEG) experiments, we show that this variability can, in part, be attributed to rhythmic light stimulation eliciting multiple, coexisting neural responses which have separable influences on behavior. Specifically, we find that rhythmic light stimulation produces neural responses at both the fundamental (*f*) and second harmonic (*2f*) frequencies, and that these responses are differentially shaped by endogenous oscillatory dynamics that vary across participants. Importantly, these harmonic responses separably contribute to perception, with gamma-band responses supporting the representation of stimulus-specific information, and alpha-band responses causally contributing to near-threshold visual perception. We reproduce these effects across datasets, paradigms, and oscillatory bands, suggesting that the multiple, concurrent oscillatory responses elicited by rhythmic light stimulation are a robust and pervasive phenomenon. We propose that the complexity of neural responses to rhythmic stimulation can explain why there is substantial variability between studies using these techniques, and that understanding these complex responses may help advance neuromodulatory technologies for both fundamental and clinical neuroscience.

## Introduction

Rhythmic light stimulation modulates brain activity (Walter and Walter 1949; Norcia et al. 2015; Hanslmayr et al. 2019; Lakatos et al. 2019), enabling us to identify causal mechanisms underpinning cognition (Herrmann et al. 2016; Bergmann and Hartwigsen 2021) and pioneer game-changing neurological interventions (Krawinkel et al. 2015; Adaikkan et al. 2019; Ichim et al. 2024). Compared to other rhythmic neuromodulatory approaches (eg transcranial magnetic stimulation [TMS]; deep brain stimulation), rhythmic light stimulation offers high clinical throughput (ie number of procedures per day) at low cost, meaning it can tackle global neurological health issues at scale. To do this, however, rhythmic light stimulation needs to be accurate (Sack et al. 2009; Wu et al. 2021) and precise (Krauss et al. 2021; Zhong et al. 2021; Soleimani et al. 2023), modulating the neural target and nothing more. Given the brain’s complex dynamics (Stam 2005; Breakspear 2017), however, we propose that such specificity is unfeasible. Instead, more success may be found in understanding the myriad neural responses elicited by a rhythmic input and accounting for how each of these responses separably influences cognition.

Neural oscillations are a common target of rhythmic light stimulation. Neural oscillations correlate with innumerable cognitive functions (Başar et al. 2001; Ward 2003; Herrmann et al. 2016), including visual perception (Busch et al. 2009; Mathewson et al. 2009), memory (Hanslmayr et al. 2016; Griffiths and Jensen 2023), and decision-making (Amemiya and Redish 2018; Samaha et al. 2020), and their disruption coincides with numerous major neurological and psychiatric disorders (Cole et al. 2017; Mably and Colgin 2018). Therefore, there is much to be gained by causally controlling oscillations. While rhythmic light stimulation has been suggested to do this (Herrmann et al. 2016), the outcomes of such studies are inconsistent: promising results (Clouter et al. 2017; Martorell et al. 2019) are followed by failures to replicate (Soula et al. 2023; Serin et al. 2024). It is unclear why these inconsistencies arise (Polanía et al. 2018), but an explanation may be found in the diversity of oscillatory responses induced by rhythmic light stimulation.

**Figure 1:**
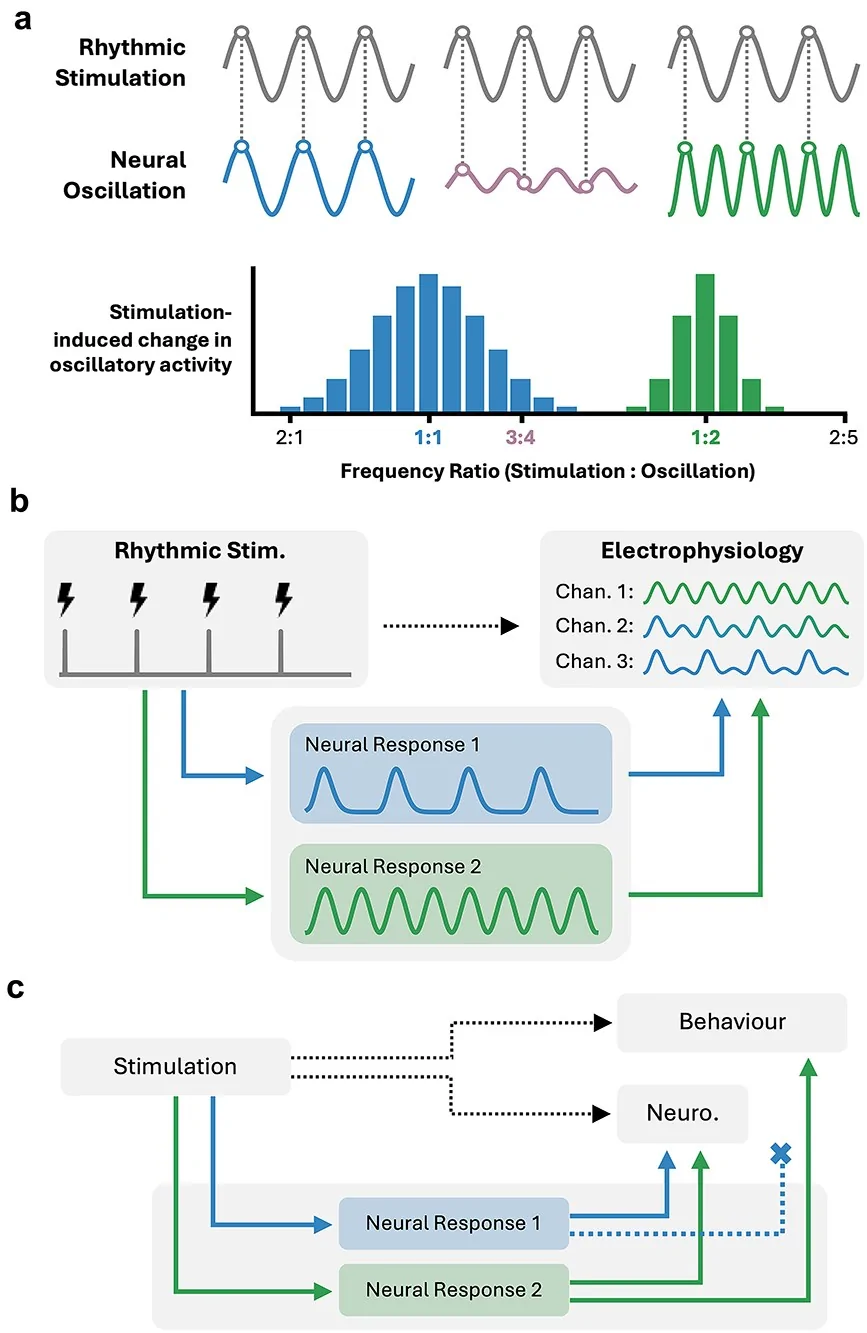
Key concepts. a) Harmonic responses to rhythmic stimulation. Rhythmic stimulation modulates oscillatory activity when the frequencies approximately align (blue) or when the oscillatory frequency is an integer multiple of the stimulation frequency (green). Stimulation frequencies between these 2 ratios (purple) do not align with the oscillatory frequency, and therefore struggle to amplify the oscillations. b) Emergence of stimulation-induced complex rhythms in electrophysiology. In this example, rhythmic stimulation produces 2 neural responses in neighboring regions: 1 at the fundamental frequency, 1 at the second harmonic. The spatial proximity of the neural responses mean they sum together and present as a complex wave on a recorded channel. None of the raw measures reflect the true neural responses (though spatial and spectral differences between the outputs can be used to reconstruct the neural responses; not visualized). c) The relationship between neural and behavioral responses to stimulation. Stimulation elicits multiple latent responses within the brain. These responses sum together to produce measurable effects in neuroimaging and behavior, but the way in which they sum together differ between measurements. In this instance, “Neural Response 1” contributes to the neural measure, but does not affect behavior. This means the raw output from 1 measure will not necessarily predict another output.

Oscillatory responses to rhythmic light stimulation are thought to be strongest when the stimulation frequency matches the resonant frequency of the target oscillation (Notbohm et al. 2016; Hanslmayr et al. 2019; Duecker et al. 2024). This principle, formalized in the concept of Arnold Tongues (Pikovsky et al. 2001), underpins much of the logic behind rhythmic brain stimulation (Notbohm et al. 2016; Hanslmayr et al. 2019; Duecker et al. 2024). However, Arnold Tongues also occur at integer multiples of the input frequency (Pikovsky et al. 2001; Jiménez et al. 2022; Fig. 1a)—a phenomenon evident throughout biology, including in tumor-suppressing proteins (Jiménez et al. 2024), chick hearts (Guevara et al. 1988), the retina of salamanders (Crevier and Meister 1998), and neural phase-phase coupling (Belluscio et al. 2012; Peylo et al. 2022). Therefore, it is plausible to suggest that oscillatory responses to rhythmic light stimulation may arise in higher harmonics as well as at the stimulation frequency, opening the door to unintentional side-effects from stimulation if these higher harmonic responses impact cognition.

To further complicate matters, neural responses to stimulation may produce complex waveshapes if the higher harmonic responses interact or summate with responses at the fundamental (Walter and Walter 1949) (eg stimulation-evoked responses at the fundamental [Capilla et al. 2011]). Take an example in which rhythmic stimulation at 4 Hz induces 2 responses: a steady-state visually-evoked response at the fundamental (*f*; 4 Hz) and an oscillatory response at the second harmonic (2*f*; 8 Hz). If the neural responses come from overlapping sources, EEG electrodes will capture a complex waveshape that reflects the weighted sum of the 4 and 8 Hz responses (see Fig. 1b). This composite signal, as captured by EEG, is not necessarily the driver of any observed change in behavior (Van Bree et al. 2025). Rather, it could be that (i) only one of the neural responses impacts behavior (see Fig. 1c), (ii) the responses have separate influences over behavior, or (iii) they interact with one another to modulate behavior. Failure to account for these possibilities will lead to erroneous interpretations of results and consequent failures to replicate. To circumvent this, we need to recover the underlying neural responses to stimulation from measured activity and relate the recovered responses to changes in cognition and/or behavior.

### Current experiment

We hypothesize that (a) rhythmic light stimulation elicits a harmonic oscillatory response that is distinct from the well-documented response at the fundamental frequency (Walter and Walter 1949; Herrmann 2001; Capilla et al. 2011; Norcia et al. 2015; Notbohm et al. 2016; Cohen and Gulbinaite 2017; Hanslmayr et al. 2019), and (b) the fundamental and higher harmonic responses have separable influences over human perception.

**Figure 2:**
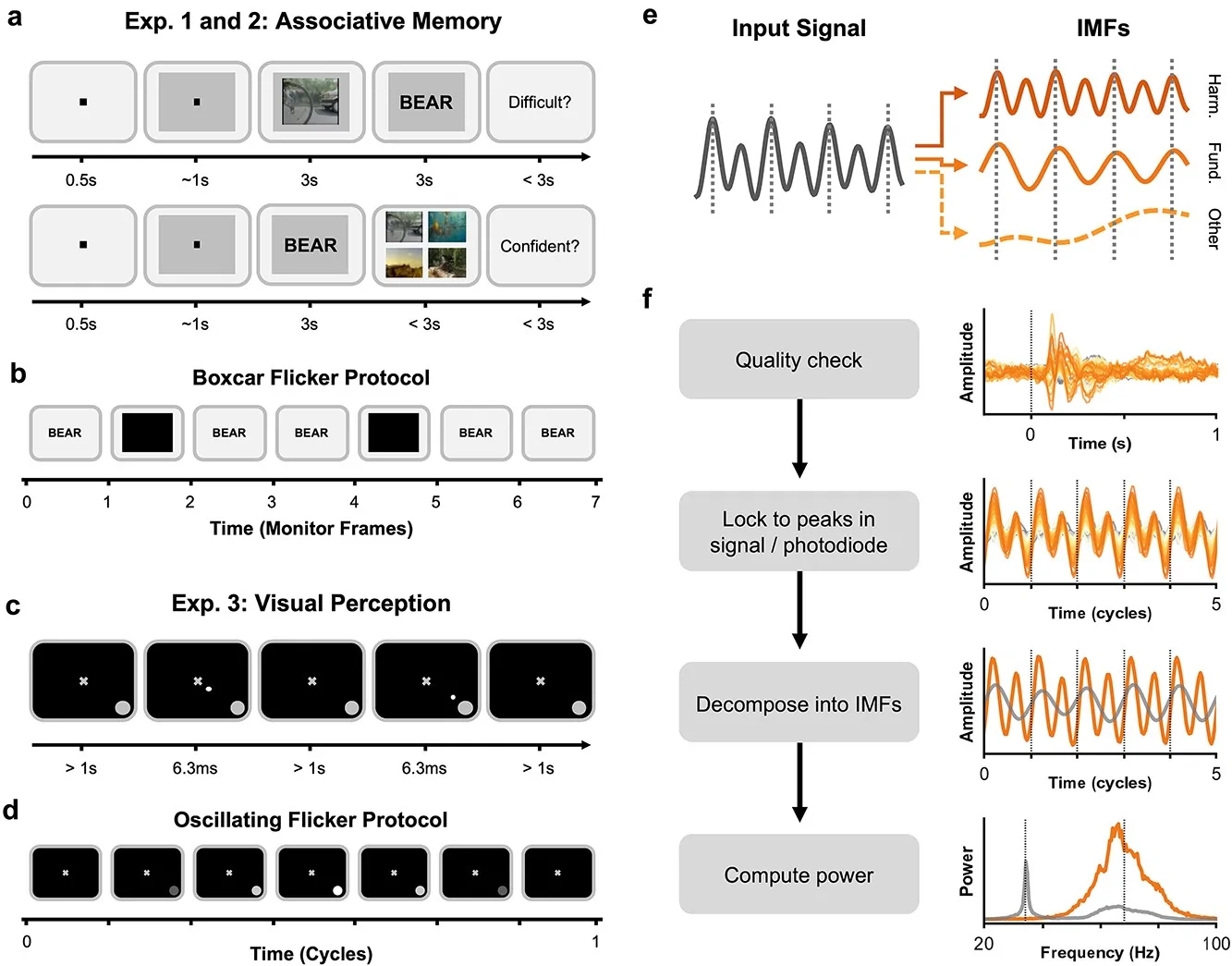
Experimental and analytical approach. a) Experiments 1 and 2 used an associative memory paradigm in which participants associated between a video (1 of 4) and a unique word. Later, they recalled the video using the word as a cue. Full details on the task/flicker are reported in Griffiths et al. (2023; bioRxiv). b) The flicker was delivered by periodically turning a patch on the screen black. The visualization describes how this was achieved for 40 Hz in experiment 1, which used a 120 Hz monitor. c) Experiment 3 used a visual perception task in which participants had to detect brief, peripheral dots while a disk oscillated in the bottom right corner. Full details are reported in Fakche and Dugué (2024, JoCN). d) One period of the oscillation reflects a transition from black to white and back to black again. e) EMD breaks a complex time series into a finite number of IMFs. We focus our analyses on the IMFs that arise at the fundamental and second harmonic stimulation frequencies. f) Pipeline for non-Fourier analysis of M/EEG data. The data undergoes standard preprocessing and quality-checking before commencing the main analyses. A peak-locked average is created using peaks in the EEG signal (exp. 1) or in a photodiode (exps 2 and 3). Solid lines indicate MEG data from 1 participant. Vertical dotted lines indicate the photodiode peaks. Peak-locking allows us to home in on the rhythmic responses to stimulation without the need for narrowband filtering (which may itself induce rhythmic effects). Peak-locking averaging does not introduce a false sense of rhythmicity (see Fig. S10). The peak-locked average is decomposed into IMFs. Gray solid line depicts the IMF at the fundamental frequency; red solid line depicts the IMF at the harmonic frequency; vertical dotted lines indicate the photodiode peaks. Spectral power is computed on the IMFs individually. Spectral power for the harmonic IMF is contrasted to a baseline condition to deduce whether harmonic power is significantly greater than what would be expected without rhythmic sensory stimulation. Gray solid line depicts power for the IMF at the fundamental frequency; red solid line depicts power for the IMF at the harmonic frequency.

Analytically, these hypotheses are challenging as standard oscillatory analyses rely on Fourier-derived power spectra (Cohen 2014), which cannot distinguish whether power increases at fundamental and harmonic frequencies reflect (i) distinct neural oscillations, or (ii) a single, non-sinusoidal oscillation (Aru et al. 2014; Cole et al. 2017; Cole and Voytek 2017; Schaworonkow 2023). This ambiguity complicates the reinterpretation of prior EEG research on harmonics. For example, while several studies (Fawcett et al. 2004; Pastor et al. 2007; Kim et al. 2011; Heinrichs-Graham and Wilson 2012; Gulbinaite et al. 2019; Retter et al. 2021) have found spatially separable harmonic components in Fourier Fast Transform (FFT) derived power spectra following rhythmic stimulation, this does not mean that rhythmic stimulation has induced a neural oscillation at the harmonic as non-sinusoidal responses to rhythmic stimulation also produce spatially separable harmonic patterns in FFT-power spectra (Fig. S1). To circumvent this problem, we use an alternative approach: empirical mode decomposition (EMD; Huang et al. 1998; Quinn et al. 2021). EMD separates the recorded electrophysiological signal into distinct rhythmic components, termed intrinsic mode functions (IMFs; Fig. 2e). Critically, overlapping oscillatory responses at the fundamental and higher harmonic frequencies will be separated as distinct IMFs if the peaks of both components are visible in the summation (for simulations, see Fig. S2A). In contrast, singular oscillations with a non-sinusoidal waveform will present as a single rhythm (Fig. S2B and C). The fundamental and harmonic IMFs we resolve can then be analyzed separately, enabling us to identify if they operate separably on a neural and/or behavioral level. To find evidence that the IMFs are statistically separable (eg spatially, temporally, or functionally) from 1 another would mean that complex waveshapes elicited by rhythmic light stimulation should not be treated as a singular entity, but rather a summation of multiple concurrent responses to stimulation.

We investigate our hypotheses in 3 datasets that paired 2 behavioral tasks (see Fig. 2a–d) with MEG/EEG recordings and rhythmic light stimulation. Using the variability in experimental protocols as a means to demonstrate the robustness of this phenomenon (see Table 1; see methods), we show that rhythmic light stimulation elicits 2 concurrent but separable neural responses, 1 at the fundamental and 1 at the second harmonic, which can be distinguished by their topographic distributions and fluctuations over time. This is replicable across tasks, electrophysiology recording modality, target frequency band, and is robust to changes in the preprocessing pipeline. Moreover, we demonstrate that the harmonic responses elucidate (a) oscillatory coding of representational content in gamma oscillations, and (b) alpha phase-dependent visual perception. Altogether, this suggests that multiple oscillatory responses to rhythmic stimulation are a pervasive and robust phenomenon, with each response uniquely impacting perception.

**Table 1:** Differences in parameters across experiments.

Parameter	Experiment 1	Experiment 2	Experiment 3
**Final sample size**	31	33	13
**Task**	Associative memory	Associative memory	Near-threshold perception
**Recording modality**	128-channel EEG	306-sensor MEG	64-channel EEG
**Stimulation frequencies**	60/40/30/24/20/17/15 Hz	68/44/33 Hz	10/8/6/4 Hz
**Stimulation waveshape**	Single frame (~2 ms)	Single frame (~7 ms)	Sinusoid
**Stimulation monitor type**	LCD monitor	ProPixx projector	ProPixx projector
**Stimulation monitor refresh rate**	120 Hz	480 Hz	480 Hz
**Referencing**	Average of montage	N/A	Mastoids
**Control**	No stimulation	Broadband flicker	Shuffled trials
**Peak identification**	EEG signal	Photodiode	Reconstruction

Differences in design across experiments enabled us to test the robustness of subharmonic stimulation across numerous potential mediating variables. This table highlights key variations (see methods for preprocessing pipelines used for each experiment).

## Materials and methods

### Experiment 1

#### Participants

Thirty-two participants were recruited (mean age = 20.1 yr; age range = 18 to 26 yr; 71.9% female; all right-handed [self-reported]). Participants were compensated with course credit or financial reimbursement. Participants provided informed consent before starting the experiment. Ethical approval was granted by the Research Ethics Committee at the University of Birmingham (reference number: ERN_18-0226AP31/ERN_18-0226AP31A).

#### Behavioral task

Participants completed a paired-associates episodic memory task, learning 192 video–word pairs across 3 blocks. During encoding, each trial began with a fixation cross (1 ± 0.2 s jitter), followed by a video clip and then an English noun (both presented for 3 s). There were 4 videos in total, each paired with 48 nouns. Videos were presented in a pseudo-randomized order such that each video would appear twice in a series of 8 trials. After encoding the video–word pair, participants reported whether they perceived the screen to be flickering during stimulus presentation. Participants had 3 s to respond using the keyboard before the next trial began.

After being exposed to all pairs of a block, participants were then subjected to a retrieval test. During retrieval, each trial began with a fixation cross (1 ± 0.2 s jitter), followed by an English noun (presented for 3 s). These nouns were taken from the immediately preceding encoding phase. Participants had 3 s to select which of the 4 videos they thought was associated with the word. Following this, a prompt asked whether the participant felt they were confident in their response. Again, participants had 3 s to provide a binary answer (“yes” or “no”).

#### Rhythmic light stimulation protocol

Rhythmic light stimulation was delivered using a 120 Hz liquid crystal display (LCD) monitor. Participants sat approximately 50 cm away from the monitor. For this, a black box was superimposed over target stimuli and fixation crosses during both encoding and retrieval. The box would appear for a single frame (8.33 ms; see Fig. 2b) of every cycle of the target frequencies (15, 17, 20, 24, 30, 40, 60 Hz). The highest stimulation frequency (60 Hz) surpassed the flicker fusion threshold and appeared as a static gray box. The control condition, where no flickering occurred, used a static gray box to perceptually match the 60 Hz condition.

There were a total of 24 trials per stimulation before preprocessing. This was sufficient to determine neural differences between stimulation conditions and the control condition, but lacked power for linking to cognition and behavior. This was remedied in experiment 2, which reduced the number of stimulation conditions to boost trial count per condition.

#### E‌EG acquisition and preprocessing

EEG was recorded from 128 Ag/AgCl active electrodes using a BioSemi Active-2 amplifier (sampling rate = 1,024 Hz). Data were preprocessed using MNE-Python (Gramfort 2013) following the FLUX pipeline (Ferrante et al. 2022) with minor adaptations for EEG. The EEG was bandpass filtered (1 to 256 Hz) and notch filtered (50, 100, 150, 200, and 250 Hz). Muscle artifacts were detected automatically using MNE’s “annotate_muscle_zscore,” identifying activity between 70 and 125 Hz exceeding a *z*-score of 10. Bad channels were identified using the random sample consensus (RANSAC) method (Bigdely-Shamlo et al. 2015). Following this, independent component analysis (ICA) was applied to remove spatially stable artifacts (eg blinks, saccades). Muscle artifact and bad channel detection were repeated after ICA to remove residual noise. Bad channels were then interpolated using the average of neighboring electrodes.

EEG data were epoched around stimulus onset (encoding: video onset; retrieval: noun onset), starting 2 s before stimulus onset and ending 2 s after stimulus offset.

#### Participant exclusion

To maximize signal-to-noise, participants who failed to show the prototypical rhythmic responses at the fundamental frequency were excluded from the main analyses. To identify these participants, for each participant and trial, Morlet wavelets were applied to the preprocessed data (width = wavelet frequency/2). SpecParam (Donoghue et al. 2020) was used to remove the aperiodic slope from the resulting power spectrum. To isolate the peak at the fundamental frequency, power at the fundamental frequency was normalized against power at ±1 Hz the fundamental frequency (eg for the 24 Hz condition, power at 23 and 25 Hz were averaged and then subtracted from power at 24 Hz). A cluster-based permutation test (permutations = 1,000; conducted across trials, for each participant individually) was used to determine whether a participant showed a reliable peak at the fundamental frequency (ie when *P* < 0.05). One participant did not pass this threshold, leaving 31 participants for further analysis.

#### Spectral separation analyses

**Figure 3:**
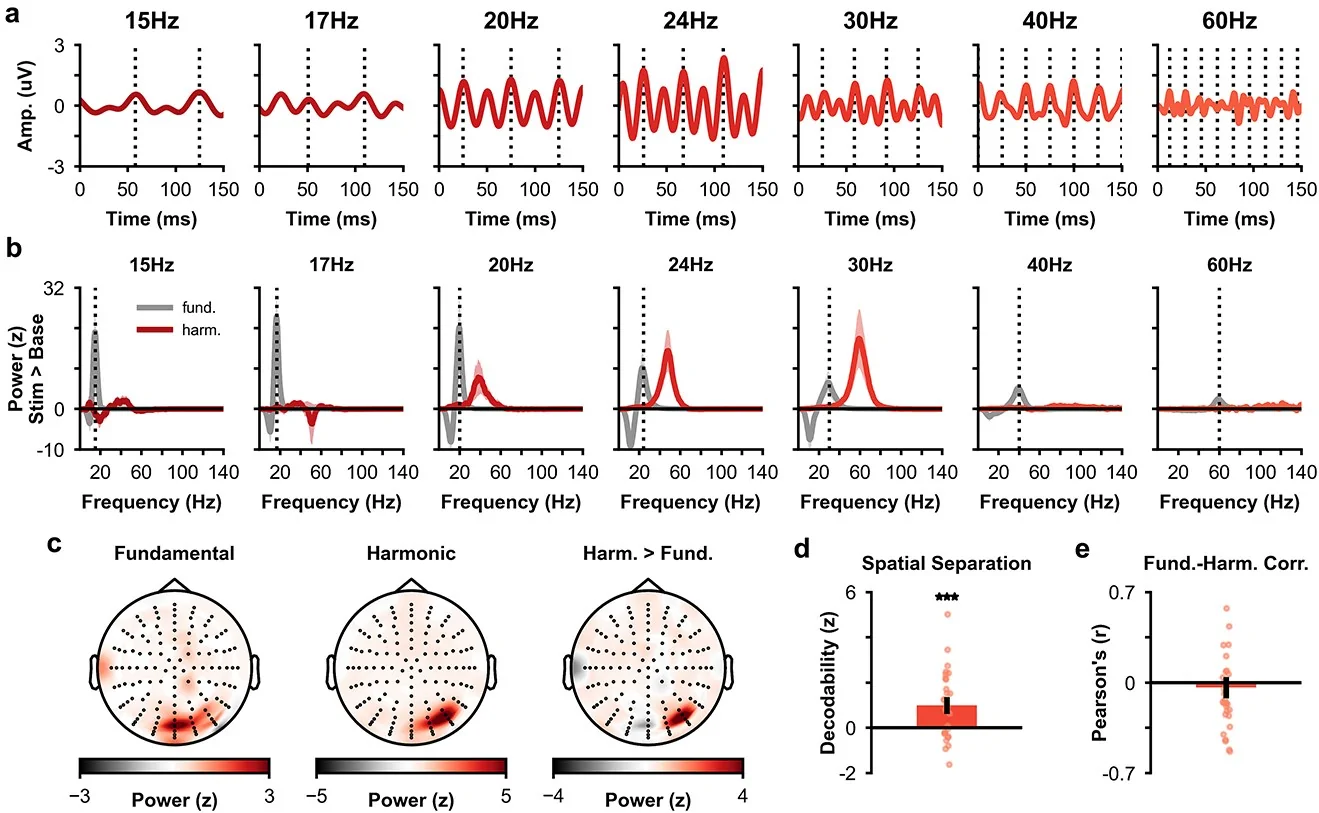
Harmonic EEG responses with the gamma-band following rhythmic sensory stimulation. a) Peak-locked average of data from a single participant for the 5 stimulation conditions. Clear harmonic responses can be seen following 20 to 30 Hz frequency stimulation, but these effects dissipate for lower (<20 Hz) and higher (>40 Hz) stimulation frequencies. Colored lines depict EEG response averaged across channels. Dotted gray lines indicate approximate time between each stimulation pulse. (b) Power for the fundamental and harmonic IMFs, averaged across participants and channels. Colored lines indicate power for the harmonic IMF; gray lines indicate power for the fundamental IMF; dotted gray line indicates stimulation frequency; shaded areas indicate standard error of the mean. c) Topography of the fundamental (left) and harmonic (middle) IMFs, centered on the fundamental and harmonic frequency respectively and then averaged across participants and conditions. The right topography depicts the difference between the 2 conditions, with positive values indicating greater power for the harmonic IMF and negative values indicating greater power for the fundamental IMF. d) The fundamental and harmonic topographies can be distinguished using linear discriminant analyses (relative to data with shuffled labels). Bar indicates group mean; dots indicate participants. e) No correlation is observed between fundamental power and harmonic power over time. Bar indicates group mean; dots indicate participants.

To visualize the complex waveforms elicited by rhythmic stimulation, EEG data were first peak-locked (Fig. 3a). Peak-locking provides spectral denoising beyond that accomplished during preprocessing, accentuating rhythmic components at the fundamental and harmonics while suppressing others. Note that peak-locking does not, in-and-of-itself, provide evidence that complex waveforms reflect the summation of separable oscillations. It does, however, enable visual verification that these waveforms contain harmonic effects and does not simply reflect non-sinusoidal oscillations at the fundamental frequency (n.b. while peak-locking could theoretically introduce an artifact where rhythms emerge at harmonics, such an artifact would appear for every harmonic [second, third, fourth, etc.] and for every stimulation condition; neither is the case in our data, suggesting our peak-locked averages reflect genuine complex neural responses). For peak-locking, the data were high-pass filtered at 12 Hz to attenuate low-frequency activity (eg endogenous alpha oscillations) and *z*-scored across time and channels to normalize scales across participants. Peaks in the EEG signal exceeding a prominence threshold of 0.5 SDs were detected and used as markers for re-epoching: each new epoch began 0.1 s after the detected peak and lasted 1 s. By excluding the first 0.1 s after the peak, we minimized aperiodic contributions to the peak-locked average. Peak-locked epochs were split by stimulation condition and averaged separately for every condition-channel pair.

EMD was then applied to the peak-locked averages to extract IMFs. For each channel, an iterated masked sift (Fabus et al. 2021) was used to extract 5 IMFs. Unlike conventional EMD, which can split a single non-sinusoidal waveform across multiple IMFs when the signal violates envelope symmetry assumptions (“mode splitting”), iterated masked sift constrains IMF frequency ranges with data-driven masks, reducing the likelihood that non-sinusoidal/asymmetric features of a waveform are erroneously split across multiple IMFs (see Fig. S2 for empirical validation). (Note that, as with any heuristic decomposition method, the separation of signal components by EMD does not, by itself, establish that the recovered components arise from distinct neural processes. Rather, the evidence for separability presented here is taken from subsequent topographic, temporal, and behavioral analyses applied to the decomposed components). We set the mask amplitude to 1 SD of the previously extracted IMF (or 1 SD of the whole signal for the first IMF extracted). We extracted 5 IMFs as this was sufficient to resolve all stimulation frequencies and separate them from lower-frequency noise (Fig. S3). Our key results did not change when resolving more IMFs. This is because, with the EMD implementation we used, any additional IMF resolved will always capture activity at frequencies below the preceding IMF, and therefore would not alter the already-resolved fundamental or harmonic IMFs used in our analyses. The instantaneous frequency, amplitude, and phase of each IMF were computed using the normalized Hilbert transform. The Hilbert–Huang transform was then used to derive the power spectrum from the instantaneous frequency and amplitude data. The fundamental IMF was defined as the IMF whose mean instantaneous frequency was closest to the stimulation frequency. The harmonic IMF was the IMF whose mean instantaneous frequency was closest to twice the stimulation frequency.

For each participant, the resulting power spectra were standardized using the SD across channels and conditions. Cluster-based permutation tests (1,000 permutations) assessed whether power at the fundamental and harmonic frequencies following stimulation differed from the baseline (ie no stimulation) condition. Clusters were considered significant when *P* < 0.05.

#### Spatial separation analyses

Here, we restricted analyses to the conditions that showed significant effects at both the fundamental and harmonic frequencies (ie 24 Hz, 30 Hz) as there was little sense in searching for spatial differences in conditions which found no evidence for multiple neural responses. Peak-locked averaging and IMF power were computed as before, but done separately for each trial rather than averaging across trials within a condition. We pooled trials across stimulation conditions to produce 2 categories: 1 for fundamental power and 1 for harmonic power.

To examine topographic differences between fundamental and harmonic responses, a 5-fold cross-validated linear discriminant analysis (LDA) was used. Four-folds formed the training set, with the fifth serving as the test set. Decision values were computed for each trial of the test set (Treder 2020), which were then averaged across trials. This process was repeated 1,000 times using shuffled labels to generate a surrogate distribution of chance-level decision values. The true decision value was then *z*-scored using the mean and SD of the surrogate distribution.

A 1-sample permutation test (1,000 permutations) was conducted to determine whether *z*-scored decision values, pooled across participants, significantly differed from the chance-level surrogate distribution (*P* < 0.05).

Note this analysis reveals whether the topographic distributions of the fundamental and harmonic responses are statistically separable, but this should not be interpreted as their spatial sources representing wholly distinct cortical regions. It remains possible, for example, that the two responses share some overlap in generating sources, or that one response emerges within a subset of the regions that generate the other source.

#### Correlations between fundamental and harmonic responses

To test whether fundamental and harmonic responses co-varied, fundamental and harmonic responses were computed per trial (as in the spatial separation analyses) and averaged across channels. A Pearson correlation was conducted across trials for each participant separately. The resulting correlation coefficients were then pooled across participants and subjected to a 1-sample permutation test. To aid interpretation of null effects, 1-sample Bayes Factor *t-*tests were used (as implemented in JASP [JASP-Team 2018]).

### Experiment 2

This experiment largely matches experiment 1. For brevity, we only describe the methods and details that differ between experiments 1 and 2.

#### Participants

A total of 37 participants were recruited (mean age = 24.7; age range = 18 to 37; 64.9% female; all right-handed [self-reported]).

#### Experimental task

See experiment 1.

#### Rhythmic light stimulation protocol

Light stimulation was delivered using a 1,440 Hz ProPixx projector at stimulation frequencies of 34, 44, and 68 Hz. Participants sat approximately 120 cm away from the monitor. In addition to a baseline condition that matched experiment 1, we included a second baseline that used a broadband 14 to 54 Hz flicker (centered on 34 Hz).

#### MEG acquisition and preprocessing

MEG was recorded using a 306-channel MEGIN Elekta Triux system, with a 1,000 Hz sampling rate. The raw data were corrected using Maxwell filters, with bad channels being marked and removed. Data were then bandpass filtered between 0.5 and 220 Hz, with notch filters at 50, 100, 150, and 200 Hz to attenuate line noise. Muscle artifacts were detected automatically using MNE’s “annotate_muscle_zscore,” identifying activity between 110 and 140 Hz that exceeded a *z*-score of 10. ICA and epoching were conducted as in experiment 1.

#### Participant exclusion

Four participants did not pass the inclusion threshold (as described in the methods for experiment 1), leaving 33 participants for further analysis.

#### Spectral separation analyses

Analyses matched those of experiment 1, with 3 exceptions.

1. Rather than detecting peaks in the MEG data itself, we used data from a photodiode that was recorded simultaneously with the MEG. The photodiode provides the flicker ground truth, so it is theoretically the better choice for peak detection.
2. Magnetometers and gradiometers were combined between computing the power spectra and conducting statistical analyses. As the magnetometers and gradiometers were already placed in a common unit space through normalization, their combination simply reflected taking the mean between the 3 sensors (1 magnetometer, 2 gradiometers) recording from the same position.
3. We contrasted the periodic stimulation conditions against the broadband stimulation baseline rather than a stimulation-free baseline. This provided us with an “active” control that rules out the possibility that light stimulation produces harmonic oscillatory responses regardless of whether the stimulation protocol itself is rhythmic or aperiodic.

#### Spatial separation analyses

Analyses matched experiment 1, with the exception that the trials used came from the 2 significant conditions: 34 and 44 Hz.

#### Correlations between fundamental and harmonic responses

See experiment 1.

#### Oscillatory coding in fundamental and harmonic responses

We applied multiclass LDA to the epochs of MEG data where participants were viewing the 4 videos. Every channel and trial was individually baseline-corrected by subtracting the mean of the amplitude between 250 and 50 ms before video onset. To speed up computation, the MEG data were downsampled to 500 Hz and re-epoched so they began 250 ms before video onset and ended 1,000 ms after video onset. Then, for each trial and sample individually, topographic patterns were *z*-scored across sensors.

The LDA classifier was trained using data from all conditions except one stimulation condition. The classifier was then tested using the held-out stimulation condition. Decision values were computed for every trial, providing an interval measure of classifier performance. This process was repeated 20 times, shuffling the labels of the training dataset to generate a surrogate distribution of the decision values expected by chance.

For time-series analysis of LDA performance, the true decision values of every participant were normalized by subtracting the mean of the surrogate distribution and dividing by the surrogate distribution’s SD. The data were then pooled and subjected to a cluster-based permutation test (1,000 permutations). LDA performance was considered significant when the resulting *P*-value was less than 0.05.

For the spectral analysis of LDA performance, we focused our analyses on activity between 100 and 400 ms post-stimulus (that is, when the classifier peaked), reasoning that samples outside of this window had little, if any, evidence of representational content. This reduced window led to unreliable EMD derivations, so we used Morlet wavelets to derive the power spectrum. Given that (i) the preceding analyses demonstrate the presence of true harmonic oscillations and (ii) the control conditions provide alternative means to separate fundamental and harmonic responses, we do not believe that this Fourier-based approach undermines the results of this section. We used wavelets at 200 equidistant frequencies between 1 and 100 Hz, all of which had a length of half of the wavelet’s frequency. As above, this process was repeated for each surrogate, with the true power spectrum being normalized frequency-by-frequency using the mean and SD of the surrogate distribution.

The resulting power spectrum produced a 1/*f* power law, including positive and negative values. Negative values cannot be easily incorporated into 1/*f* correction procedures, so rather than removing the 1/*f*, we computed the change in power at the target frequencies (34 and 68 Hz) relative to neighboring frequencies (ie ±5 Hz). Decoding performance at the target frequency was directly compared to its neighbors in a permutation-based *t-*test (1,000 permutations). A peak was considered significant if it was significantly larger than the neighboring frequencies and the *P*-value was less than 0.05.

These steps were repeated for 2 control analyses using bandstop filters. The first control suppressed activity at the fundamental frequency using infinite impulse response filters with sidebands at ±10 Hz. The second control suppressed the second harmonic activity, using infinite impulse response filters with sidebands at ±10 Hz. All other aspects of the analysis remained the same.

### Experiment 3

We re-analyzed an existing, preprocessed dataset of 17 participants. An outline of the experimental paradigm and stimulation protocol is presented in Fig. 2c and d. For further details, see the original paper (Fakche and Dugué 2024). The experimental approach and stimulation parameters differ from experiments 1 and 2 as experiment 3 was an independent project running in tandem with experiments 1 and 2. Only after observing the effects of experiments 1 and 2 did we realize that the data from experiment 3 could act as a conceptual replication. As all data had been collected by this point, we could not adapt the experimental parameters to better align with experiments 1 and 2. We do not consider this a limitation; rather, the differences provide an opportunity to assess the robustness of harmonic responses to stimulation under varying experimental conditions and parameters.

#### Participant exclusion

Four participants did not pass the inclusion threshold (as described in the methods for experiment 1), leaving 13 participants for further analysis.

#### Spectral separation analyses

Analyses matched that of experiment 1, with 2 exceptions.

1. We reconstructed a photodiode from EEG triggers and used this to detect peaks in the data.
2. This dataset lacked a baseline condition that involved participants performing the task. Therefore, we compared the stimulation conditions to the eyes-open resting state (see original paper for details).

#### Spatial separation analyses

Analyses matched experiment 1, with the exception that the trials came from the 3 significant conditions: 4, 6, and 8 Hz.

#### Correlations between fundamental and harmonic responses

See experiment 1.

#### Individual difference analyses

Individual alpha frequency (IAF) was computed using eyes-open resting state data. EMD was used to estimate the power spectra of the preprocessed EEG. The approach matched that used for the spectral separation analyses, with the exception that we computed power across all IMFs rather than selecting IMFs at the fundamental/harmonic frequencies—this change circumvented the need to make assumptions about the approximate frequency of the endogenous alpha oscillation. IAF was quantified as the largest peak in the average power spectrum for the posterior central channels (Oz, O1, O2, POz, PO3, PO4, Pz, P1, P2).

To compute correlations between IAF frequency and 6 Hz fundamental/12 Hz harmonic responses, we took the fundamental/harmonic responses used for the spectral separation analyses, averaged these responses across channels, and restricted them to the relevant frequency (that is, the fundamental power spectrum was restricted to power at 6 Hz; the harmonic power spectrum was restricted to 12 Hz). Pearson’s correlations were conducted across participants, for the fundamental and harmonic responses separately. The correlation was considered significant when the *P*-value was less than 0.05.

#### Analyses linking visual perception to fundamental and harmonic neural responses

We began by modeling perceptual performance as a function of luminance change and its second harmonic. To this end, we generated a time series of 100 samples that covered a single flicker cycle. For every sample, we identified trials with stimuli presented at that flicker phase (±0.1π) and computed the mean performance across these trials.

Two sine waves were generated; 1 at the fundamental flicker frequency and the other at the second harmonic. The phase of the harmonic sine was at a fixed offset of −0.5π relative to the fundamental so the peaks of the fundamental and harmonic frequencies aligned. We used this approach as this led to model waveshapes that best resembled the complex waveshapes we observed in the EEG data. For an exploration of how the model fits change as a function of relative phase between the fundamental and harmonic sine waves, see Fig. S4. For each participant, these were fitted to the perceptual performance time course in 2 models. The first model consisted of a constant and the fundamental frequency sine wave only. The second consisted of a constant and both sine waves. Akaike information criterion (AIC) and Bayesian information criterion (BIC) were computed for both models. The changes in AIC/BIC between models were pooled across participants and then subjected to a 1-tailed, 1-sample *t*-test. Model improvement was deemed significant when the AIC/BIC was consistently lower than zero across participants and the associated *P*-value was less than 0.05. We treat AIC as our primary metric for adjudicating between these models, as AIC evaluates which candidate model better approximates the data, whereas BIC evaluates which candidate is the true generating model (Burnham and Anderson 2004). Since neither model accounts for all neural processes influencing perceptual behavior (eg errors in motor responses are not accounted for), the assumptions of BIC are not well-suited to this comparison. Nonetheless, we report both measures for completeness.

To link this back to the complex waveforms observed in the EEG, for each channel individually, we took the first cycle of the fundamental IMF and the first 2 cycles of the harmonic IMF (to match the duration to the fundamental IMF) of the peak-locked average. These waveforms were interpolated to match the 100 sample-long time-course of the behavioral data, *z*-scored, and then used in place of the sine waves in the analysis above. Cluster-based permutation tests (permutations = 1,000) were used to assess whether power AIC/BIC was consistently lower than zero across participants (ie *P* < 0.05).

### Limitations of EMD

EMD is an emerging trend for M/EEG analysis, and best practices are still being established. As such, there is no consensus on how best to tackle limitations of EMD. We highlight several key limitations here, together with how we mitigated them.

1. There is no formulation to determine the number of IMFs to extract. To address this, we constructed our own data-driven criterion. This involved extracting the IMFs required to resolve the slowest stimulation frequency, plus 1 additional IMF to separate this from lower-frequency activity (see Fig. S3), ensuring we had all necessary IMFs for our analysis. Increasing the number of extracted IMFs beyond this threshold had no impact on the significance of any reported results.
2. EMD is susceptible to mode mixing, where spectrally-adjacent components are summed together in a single IMF, particularly when noise is high. We minimized this risk by first computing peak-lock averages to boost the signal-to-noise ratio of oscillatory components of the signal, and then applying a masked sift (Fabus et al. 2021), which uses a spectral mask to separate neighboring spectral components before running EMD. Together, these approaches drastically reduce the risk of mode mixing in our analyses. Moreover, it is worth noting that, in the context of these experiments, mode-mixing would result in the failure to separate responses (a false negative, rather than a false positive); meaning mode mixing would understate rather than exaggerate the prevalence of multiple responses to rhythmic stimulation.
3. The masked sift introduces free parameters around the choice of mask frequencies. As with the IMF issue above, we mitigated this issue by taking a data-driven approach to selecting mask frequencies (ie the “iterated masked EMD”; Fabus et al. 2021). Though we cannot say for certain that the data-driven decisions produce the optimal masks, the consistency of our results across 3 experiments with different stimulation frequencies, recording modalities, and preprocessing pipelines suggests that the decomposition is not unduly sensitive to these choices.
4. The masked sift also requires a mask amplitude parameter, which determines the amplitude of the sinusoidal mask added to the signal before decomposition. Here, we used the toolbox default, which is 1 SD of the previously extracted IMF (or 1 SD of the whole signal for the first IMF extracted). Simulations confirmed that this value lies within a broad region of reliable separation for summed oscillations at *f* and 2*f* (Fig. S5). Separation failed only when the harmonic amplitude was substantially weaker than the fundamental (a problem that impacted all tested mask amplitudes, indicating a signal-detection floor rather than a problem specific to using the default parameter). Critically, no mask amplitude value produced spurious harmonic separation from a single oscillation, suggesting that cases where EMD recovers a harmonic component reflect genuine spectral content rather than decomposition artifacts. Taken together, this suggests that iterated mask EMD is conservative rather than permissive when resolving harmonic components to rhythmic stimulation. Notably, when systematically varying the mask amplitude applied to the acquired data, we did not observe any change in the significance of any reported spectral separation result, with the exception of a single condition at the most extreme setting tested (30 Hz fundamental, mask amplitude = 5, experiment 1; Fig. S6).
5. More generally, EMD (including the iterated mask EMD) is a heuristic decomposition method. The separation of a signal into IMFs does not constitute evidence that the recovered components arise from distinct neural processes: independent oscillations can be merged if masking suppresses their peaks, and dependent oscillations can be separated if their instantaneous frequencies are sufficiently distinct. In the present study, we treat EMD as a necessary precondition for assessing the separability of fundamental and harmonic responses, rather than evidence of separability itself, with the case for separability resting on converging evidence derived from subsequent topographic, temporal, and behavioral analyses.

It is worth noting that while alternative approaches to distinguishing fundamental and harmonic responses do exist (eg higher-order spectral methods such as bicoherence; Dumermuth et al. 1971; Sheremet and Qin 2025), we elected to use EMD because it recovers each oscillatory component as a time series, enabling direct visual inspection of co-occurring fundamental and harmonic responses on a trial-by-trial and channel-by-channel basis (eg Fig. 3a). This means that we could directly relate our results to visible, interpretable features in the data through every step of the pipeline, bolstering confidence in our interpretations in the end statistics.

## Results

### Rhythmic light stimulation elicits multiple oscillatory responses that are spectrally, spatially, and temporally separable

We began by asking whether beta-band (20 to 30 Hz) stimulation elicits concurrent oscillatory responses at its fundamental (20 to 30 Hz) and second harmonic (40 to 60 Hz; ie gamma) frequencies. Participants completed an associative memory task while undergoing scalp EEG recordings and rhythmic light stimulation. We stimulated at 60, 40, 30, 24, and 20 Hz, and included a baseline condition without stimulation. Then, using EMD to unmix overlapping oscillatory signals in the EEG, we focused our analyses on (a) the IMF that most closely matched the stimulation frequency (*f*Hz; from here on, the “fundamental”) and (b) the IMF that most closely matched the second harmonic (*2f*Hz; the “harmonic”) (see methods for full details on the approach to EMD). We used the Hilbert–Huang transform to compute the power spectrum for the 2 IMFs and then compared these power spectra to their equivalents in the baseline condition.

Rhythmic light stimulation significantly increased fundamental power relative to baseline (fundamental; 15 Hz, *z* = 16.96; *P* < 0.001; 17 Hz: cluster *z* = 15.87; *P* < 0.001; 20 Hz: cluster *z* = 24.96; *P* < 0.001; 24 Hz: cluster *z* = 16.28; *P* < 0.001; 30 Hz: cluster *z* = 9.31; *P* < 0.001; 40 Hz: cluster *z* = 25.80; *P* < 0.001; 60 Hz: cluster *z* = 12.82; *P* < 0.001; see Fig. 3a and b). Critically, beta-band stimulation frequencies also led to a significant increase in harmonic power relative to baseline (stimulation at 20 Hz, response at 40 Hz: cluster *z* = 1.45; *P* = 0.043; stimulation at 24 Hz, response at 48 Hz: cluster *z* = 7.48; *P* = 0.002; stimulation at 30 Hz, response at 60 Hz: cluster *z* = 8.13; *P* = 0.001). These results demonstrate that beta-band rhythmic stimulation produces oscillatory responses at fundamental and harmonic effects that are spectrally separate.

However, harmonics for slower (<20 Hz) or faster (>30 Hz) stimulation frequencies did not reach significance (stimulation at 15 Hz, response at 30 Hz: cluster *z* = −0.64; *P* = 0.787; stimulation at 17 Hz, response at 34 Hz: cluster *z* = −0.47; *P* = 0.758; stimulation at 40 Hz, response at 80 Hz: cluster *z* = −0.30; *P* = 0.557; stimulation at 60 Hz, response at 120 Hz: cluster *z* = −1.07; *P* = 0.878). Taken together with the positive harmonic results above, these produces an Arnold Tongue-like pattern where rhythmic harmonic responses are strongest between 40 and 60 Hz (stimulation between 20 and 30 Hz), and weaken on either side of this band. Parenthetically, the absence of significant harmonic effects in the slowest and fastest conditions argues against the possibility of signal processing decisions (eg filtering, re-referencing) artificially introducing the second harmonic during data analysis.

To determine whether these responses are spatially separable, we used cross-validated LDA. The classifier was trained to categorize IMF envelopes (fundamental vs. harmonic) using channels as features. We applied the trained classifier to a held-out fold and computed decision values (as in Linde-Domingo et al. 2019). We normalized the result against a surrogate distribution generated by repeating this pipeline 20 times with shuffled category labels. This classifier performed significantly better than chance (*z* = 4.58, *P* < 0.001; see Fig. 3c and d), indicating that the fundamental and harmonic IMFs have distinct topographies.

To determine whether the fundamental and harmonic responses are temporally separate, we correlated the envelopes of the fundamental and harmonic IMFs over trials. We found no statistically significant correlation (*z* = −2.33, *P* = 0.542 s; BF_10_ = 0.229, “moderate” evidence for null [Van Doorn et al. 2021]; see Fig. 3e). To confirm that this null result reflects genuine separability rather than insufficient trial-to-trial variability in the signal (leaving measurement noise to drive the null correlation), we assessed the split-half reliability of each IMF. Both showed strong reliability (fundamental IMF: *r*_xx_ = 0.793, *z* = 29.93, *P* < 0.001; harmonic IMF: *r*_yy_ = 0.793, *z* = 29.93, *P* < 0.001), suggesting that the lack of a correlation between IMFs is better explained by the fundamental and harmonic IMFs fluctuating independently over time than measurement noise suppressing the correlation. Paired with the topographic analyses above, this suggests that the fundamental and harmonic responses have distinct neural origins.

Altogether, the results from experiment 1 suggest that beta-band stimulation produces harmonic gamma-band responses that are spectrally, spatially, and temporally separable from responses at the fundamental frequency.

**Figure 4:**
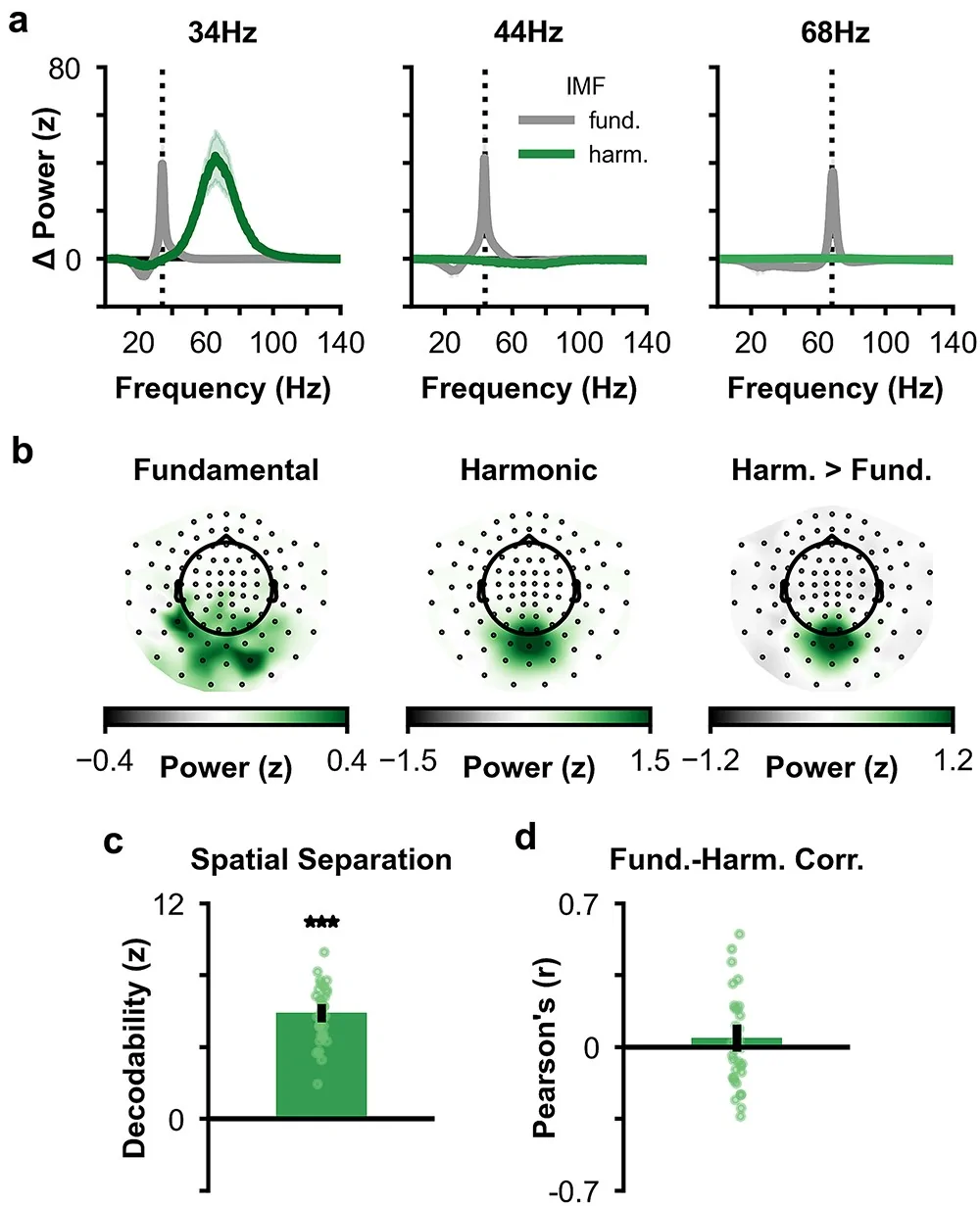
Conceptual replication of harmonic responses in gamma-band MEG and alpha-band EEG data. a) MEG power for the fundamental and harmonic IMFs, averaged across participants and channels. Colored lines indicate power for the harmonic IMF; gray lines indicate power for the fundamental IMF; dotted gray line indicates stimulation frequency; shaded areas indicate standard error of the mean. b) Topography of the fundamental (left) and harmonic (middle) IMFs, centered on the fundamental and harmonic frequency respectively and then averaged across participants and conditions. The right topography depicts the difference between the 2 conditions, with positive values indicating greater power for the harmonic IMF and negative values indicating greater power for the fundamental IMF. c) The fundamental and harmonic topographies can be distinguished using linear discriminant analyses (relative to data with shuffled labels). Bar indicates group mean; dots indicate participants d) No correlation is observed between fundamental power and harmonic power over time. Bar indicates group mean; dots indicate participants. e) See panel a. f) See panel b. g) See panel c. h) See panel d. (***P < 0.001).

We conceptually replicated these results in an independent MEG dataset, which used the same task and similar stimulation frequencies (34, 44, and 68 Hz; see Fig. 4). As above, we found that the slower stimulation frequencies produced significant harmonic responses within the gamma band (stimulation at 34 Hz, response at 68 Hz: cluster *z* = 13.37, *P* < 0.001; stimulation at 44 Hz, response at 88 Hz: cluster *z* = 8.61, *P* = 0.003) while the fastest stimulation frequency did not (stimulation at 68 Hz, response at 136 Hz: cluster *z* = 0.15, *P* = 0.279). Aligning with experiment 1, the 68 Hz harmonic response to 34 Hz stimulation was spatially and temporally separable from the 34 Hz response (spatial LDA analysis: *z* = 22.86, *P* < 0.001; temporal correlation: *z* = 0.272, *P* = 0.345, BF_10_ = 0.291 [“moderate” evidence for a lack of correlation between fundamental and harmonic responses, Van Doorn et al. 2021]). As in experiment 1, split-half analyses revealed strong reliability (fundamental IMF: mean r_xx_ = 0.75, *z* = 28.35, *P* < 0.001; harmonic IMF: mean r_yy_ = 0.89, *z* = 31.41, *P* < 0.001), suggesting that the lack of a temporal correlation between IMFs is unlikely to be due to measurement noise suppressing a correlation.

Altogether, these results conceptually replicate those of experiment 1, demonstrating that fundamental and harmonic gamma-band responses to rhythmic light stimulation are spectrally, spatially, and temporally separable from beta-band responses at the fundamental frequency.

### Rhythmic stimulation facilitates oscillatory coding in harmonic responding frequencies

If harmonic gamma-band responses reflect changes in endogenous gamma oscillatory activity, these harmonic responses should influence the neural phenomena associated with gamma oscillations. One such phenomenon is oscillatory coding, where incoming visual information is represented in specific phases of gamma oscillations (Thut et al. 2012; Lisman and Jensen 2013; Fries 2015; Watrous et al. 2015). To test this idea, we conducted LDA on the MEG data collected while participants were viewing dynamic visual stimuli (see Fig. 2a), training the classifier to discriminate the 4 videos using data from all conditions except 34 Hz and applied the classifier to the held-out 34 Hz data. We then transformed the resulting decoding time series into the frequency domain (as in Kerrén et al. 2018, 2023) to determine whether visual information is coded in the harmonic gamma-band responses.

**Figure 5:**
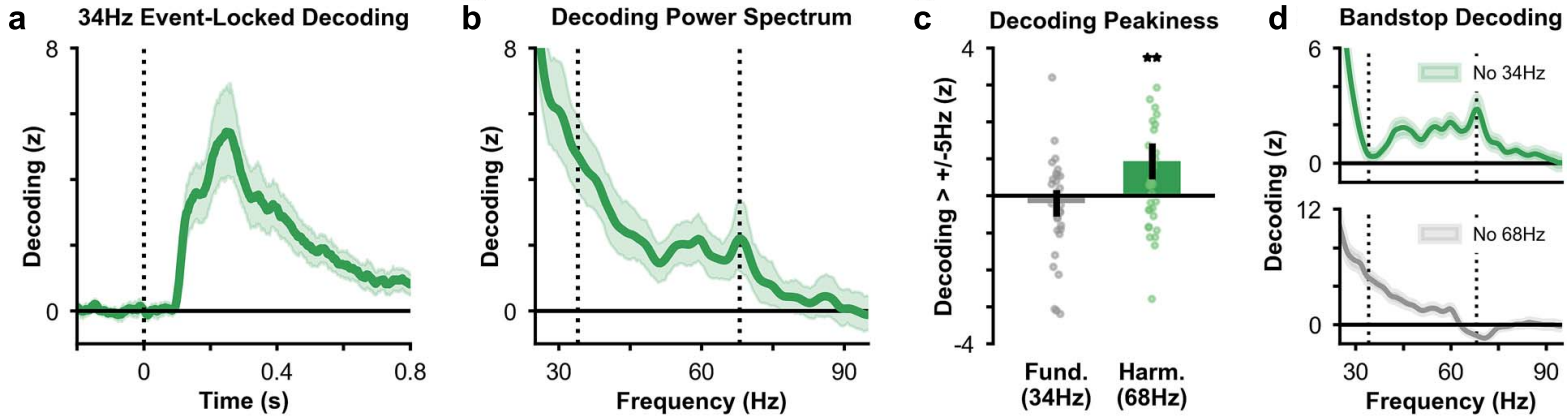
Oscillatory coding of visual content in gamma oscillations. a) Decoding performance locked to stimulus onset (time = 0). Decoding performance reflects the distance to the decision boundary, scaled by the mean and standard deviation of label-permuted surrogate data (1,000 permutations). Solid green line indicates mean performance across participants; shaded area reflects standard error of this mean. b) Decoding performance was transformed to the frequency domain on a trial-by-trial basis and then averaged across participants and trials. Solid green line indicates mean performance across participants; shaded area reflects standard error of this mean. c) Decoding power at 68 Hz (the second harmonic) showed significantly greater decoding than neighboring frequencies at 63/73 Hz. No similar effect was observed at 34 Hz (the fundamental frequency of stimulation). d) The analyses in panels b and c were repeated on bandstop-filtered data at 34 Hz (top) and 68 Hz (bottom). The 68 Hz effect persists when using a 34 Hz bandstop, but not a 68 Hz bandstop.

We first set out to confirm that we could decode visual content in the time-series MEG data. To do so, we compared the time series of LDA decoding to a surrogate distribution built from shuffling video labels, finding that the true time series showed significantly greater decoding than what would be expected by chance (*z* = 27.85, *P* < 0.001; see Fig. 5a). Then, to pinpoint an oscillatory code, we converted the decoding time series to the frequency domain and looked for rhythmic fluctuations in representational content. When comparing decoding at the harmonic frequency relative to its neighbors (±5 Hz), we observed a significant peak in decoding (*z* = 2.48, *P* = 0.017; see Fig. 5b and c). We observed no similar effect at 34 Hz (*z* = −0.54, *P* > 0.5), with the Bayes Factor revealing moderate evidence for the null (BF_10_ = 0.278). This suggests that the harmonic gamma-band responses to stimulation rhythmically represent incoming visual information, whereas responses at the fundamental frequency (ie beta-band) do not.

To rule out the possibility that this effect reflects amplitude differences at 34 Hz (Higgins et al. 2022), we ran 2 control analyses. First, we bandstop-filtered around 68 Hz, reasoning that if 34 Hz activity were driving the effect, it should persist when 68 Hz activity has been removed. Doing so, however, removed the 68 Hz peak in decoding (*z* = −5.15, *P* > 0.5, BF_10_ = 0.062; see Fig. 5d), suggesting that 34 Hz is not driving the effect. Second, we bandstop-filtered around 34 Hz to remove potential contributions of 34 Hz to the 68 Hz condition. Here, the peak at 68 Hz remained significant (*z* = 2.98, *P* = 0.006; see Fig. 5d), further suggesting that 34 Hz is not contributing to the effect.

These results demonstrate that gamma-band harmonic responses to rhythmic stimulation support oscillatory coding of incoming visual information in a manner distinct from responses at the fundamental frequency.

### Rhythmic light stimulation elicits multiple oscillatory responses in the theta- and alpha-bands

**Figure 6:**
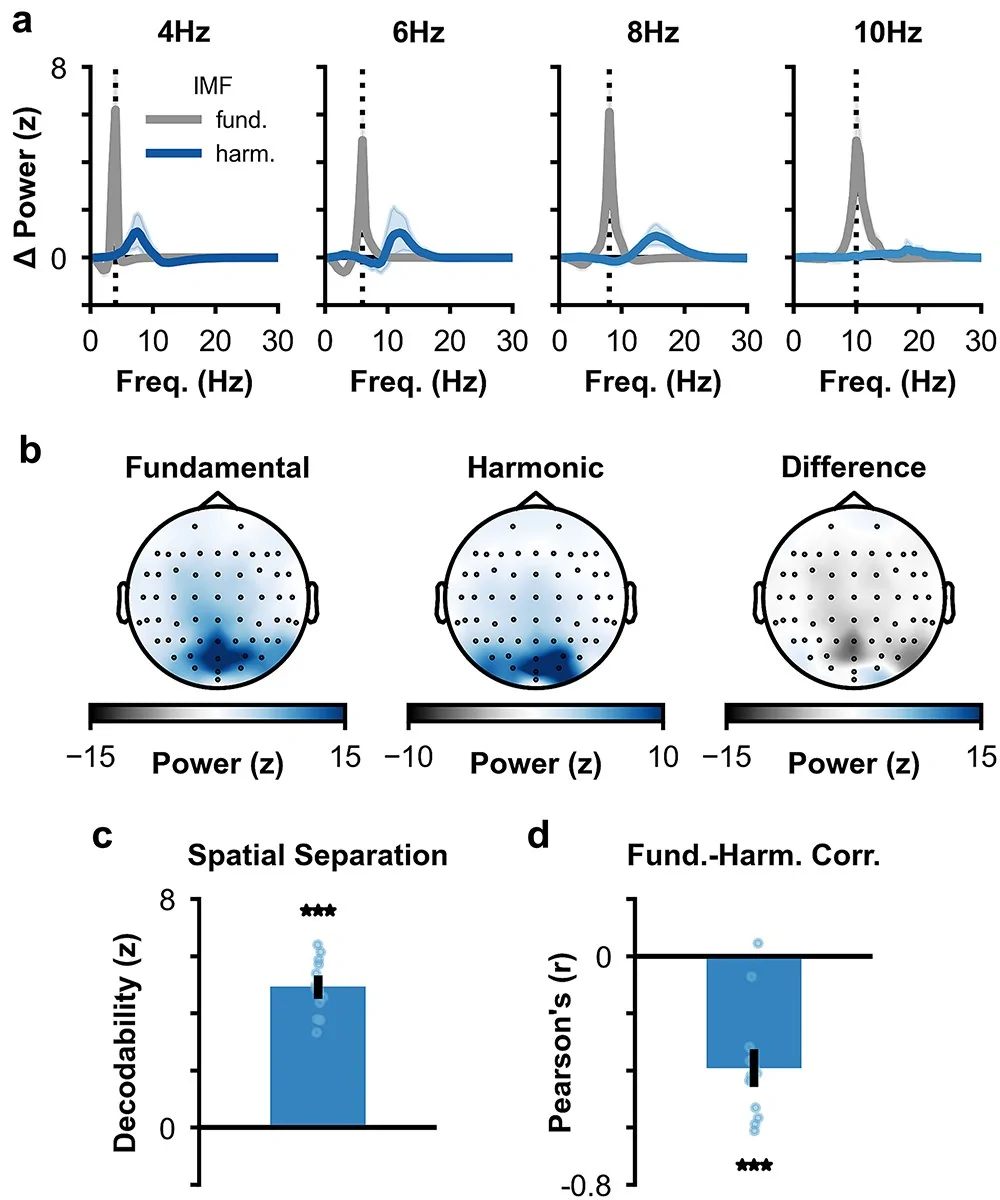
Harmonic responses in alpha-band EEG data. a) EEG power for the fundamental and harmonic IMFs, averaged across participants and channels. Colored lines indicate power for the harmonic IMF; gray lines indicate power for the fundamental IMF; dotted gray line indicates stimulation frequency; shaded areas indicate standard error of the mean. b) Topography of the fundamental (left) and harmonic (middle) IMFs, centered on the fundamental and harmonic frequency respectively and then averaged across participants and conditions. The right topography depicts the difference between the 2 conditions, with positive values indicating greater power for the harmonic IMF and negative values indicating greater power for the fundamental IMF. c) The fundamental and harmonic topographies can be distinguished using linear discriminant analyses (relative to data with shuffled labels). Bar indicates group mean; dots indicate participants. d) A significant negative correlation is observed between fundamental power and harmonic power over time. Bar indicates group mean; dots indicate participants (***P < 0.001).

Confident that beta-band stimulation can reliably induce harmonic responses in the gamma-band that are spatially, temporally, and functionally distinct from responses at the fundamental, we set out to generalize this phenomenon to theta-band stimulation and visual alpha oscillations. We reanalyzed data from a recent visual perception study (Fakche and Dugué 2024) in which participants underwent EEG while a disk oscillated in luminance on the periphery of participants’ vision (at 4, 6, 8, and 10 Hz; see Fig. 2c and d). Taking the same approach as in experiments 1 and 2, we found that rhythmic light stimulation significantly increased fundamental power relative to baseline (4 Hz: cluster *z* = 12.65, *P* = 0.002; 6 Hz: cluster *z* = 17.63, *P* < 0.001; 8 Hz: cluster *z* = 14.69, *P* = 0.002; 10 Hz: cluster *z* = 15.71, *P* < 0.001; see Fig. 6a). Critically, slower stimulation frequencies produced harmonic responses (stimulation at 4 Hz, response at 8 Hz: cluster *z* = 2.51, *P* = 0.028; stimulation at 6 Hz, response at 12 Hz: cluster *z* = 2.97, *P* = 0.023; stimulation at 8 Hz, response at 16 Hz: cluster *z* = 2.81, *P* = 0.021), whereas faster stimulation at 10 Hz did not (stimulation at 10 Hz, response at 20 Hz: cluster *z* = −0.296, *P* = 0.517). The lack of a 20 Hz harmonic response to 10 Hz stimulation might seem to contradict past research (Herrmann 2001; Pastor et al. 2007), but we believe this is simply due to a difference in methodology. Previous studies used the FFT to assess harmonic frequencies, meaning the harmonic response could reflect non-sinusoidal properties of the 10 Hz oscillation (rather than a true 20 Hz oscillation). Our use of EMD sidesteps this issue, and given that we do not observe a significant harmonic response, this suggests that harmonic results in these previous studies may be better explained as reflection of the non-sinusoidal properties of the 10 Hz oscillation rather than a true 20 Hz oscillation.

As with experiments 1 and 2, the fundamental and harmonic IMFs were spatially separable (*z* = 18.74, *P* = 0.001; see Fig. 6b and c). Intriguingly, however, we observed a significant negative correlation between time courses (*z* = −10.94, *P* = 0.002; see Fig. 6d), suggesting that harmonics were strongest when the fundamental response was weakest.

These results, paired with the gamma-band responses above, demonstrate that fundamental and harmonic responses to rhythmic stimulation are replicable and generalizable across frequency bands. Therefore, we suggest that multiple concurrent oscillatory responses to rhythmic input are a pervasive phenomenon in rhythmic stimulation research.

### IAF predicts participant-specific responses at fundamental and harmonic frequencies

Synchronization theory states that harmonic responses to rhythmic stimulation arise when there is sufficient alignment between the endogenous oscillatory frequency and an integer multiple of the input frequency (Pikovsky et al. 2001). Given that endogenous oscillatory frequency differs between individuals, this begs the question: do differences in IAF explain individual differences in harmonic alpha responses to rhythmic light stimulation?

**Figure 7:**
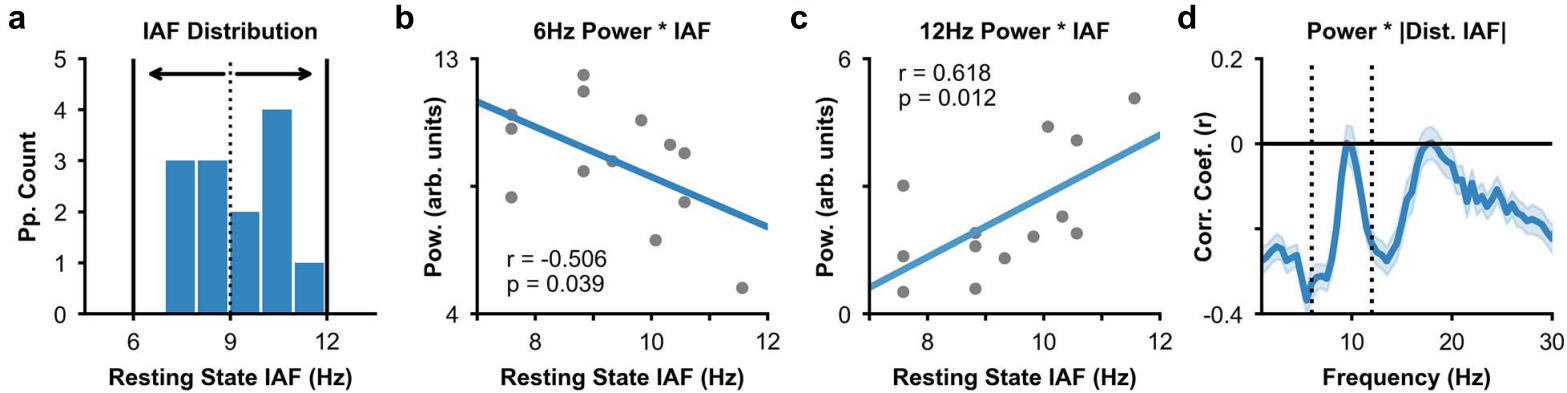
Across-participant variability in harmonic responses relates to IAF. a) IAF for each participant was calculated using eyes-open resting state data. Following the notion of Arnold Tongue, we hypothesized that 6 Hz sensory stimulation would produce a strong response at the fundamental frequency for those with a slower IAF and a greater response at the harmonic (12 Hz) for those with a faster IAF. b) We find a negative correlation between IAF and power for the fundamental IMF following 6 Hz, indicating that power at the fundamental frequency decreases as IAF increases. Conversely, we find a significant positive correlation between IAF and power at the harmonic IMF, indicating that harmonic power increases as IAF increases. Dots indicate individual participants. Lines reflect line-of-best-fit computed using least-squares regression. c) Magnitude of correlation between power and the absolute distance to the IAF across all frequencies. At both 6 and 12 Hz, there is a significant negative correlation indicating that participants with IAFs further away from 6/12 Hz show reduced power at these frequencies. d) There is no effect at intermediate alpha frequencies (ie 7 to 11 Hz) indicating that this is not a trivial correlation between IAF and alpha power but an effect specific to the fundamental and harmonic frequencies of 6 Hz stimulation. Dark blue line indicates mean correlation coefficient across channels; shaded area indicates standard error of correlation coefficient across channels.

We began by extracting participants’ IAFs using eyes-open resting state data (see methods). As no rhythmic stimulation was applied during the resting-state recordings, this means we sidestep the difficulties in extracting IAF that occur when using data involving concurrent stimulation (Keitel et al. 2019). As the IAFs of the sample were approximately evenly distributed around 9 Hz (see Fig. 7a), we focused our analyses on the 6 Hz stimulation condition to observe a potential interaction across participants where participants with slower IAFs (<9 Hz) produce stronger effects at the fundamental (6 Hz) and those with faster IAFs (>9 Hz) produce stronger effects at the harmonic (12 Hz).

When participants underwent 6 Hz stimulation, we observed a significant positive correlation between IAF and 12 Hz power (Spearman’s *ρ* = 0.618, *P* = 0.012), indicating that participants with faster IAFs produced stronger harmonic responses. Conversely, when correlating IAF and 6 Hz power, we observed a negative correlation (Spearman’s *ρ* = −0.506, *P* = 0.039; see Fig. 7b and c), indicating that participants with slower IAFs produced stronger responses at the fundamental. No similar effects were observed for power at frequencies other than the fundamental or harmonic (see Fig. 7d). These results indicate that harmonic responses to rhythmic light stimulation are strongest when the second harmonic of the rhythmic input matches the fundamental of the endogenous oscillatory frequency.

To determine whether the link between IAF and harmonic responses generalizes beyond the 6 Hz condition, we computed the absolute distance between each participant’s IAF and the stimulation frequency and correlated this with the magnitude of the harmonic response across participants (see Fig. S7). We switched to using the absolute distance to the IAF to facilitate interpretation of the correlations; now, the directionality of the correlation should always be negative (ie response magnitude decreases as the distance to IAF increases). We observed significant negative correlations for the 6 Hz (12 Hz response; *r* = −0.639, *P* = 0.009) and 8 Hz (16 Hz response; *r* = −0.573, *P* = 0.020), aligning with Arnold Tongues. Furthermore, for the 10 Hz condition, which did not produce a significant harmonic response, we did not observe a correlation (*r* = 0.424, *P* = 0.925). However, we also did not find a correlation for the 4 Hz condition (8 Hz response; *r* = 0.160, *P* = 0.700). This is harder to square with Arnold Tongues, but may be attributable to (i) limited between-participant variability in IAF distance compared to other conditions, reducing statistical power, or (ii) a genuine null effect where, given that all IAFs are relatively close to 8 Hz, the harmonic responses may be uniformly strong, with residual variability between participants being driven by other factors. Altogether, these results suggest that individual differences in IAF shape harmonic responses to rhythmic stimulation, though further investigation is warranted to understand why effect sizes vary between stimulation conditions.

The absence of resting state data for the other datasets precluded similar analyses for individual gamma frequencies. However, descriptively speaking, we can observe a related phenomenon in experiment 1 when stimulating at lower frequencies (ie 20 Hz). Given that the second harmonic (40 Hz) sits at the lower bounds of the canonical visual gamma band (~30 to 80 Hz [Buzsáki and Wang 2012; Hermes et al. 2015a; Griffiths and Jensen 2023]), we might expect participants with fast endogenous gamma to show responses at higher harmonics (eg 3*f*, 60 Hz). Visual inspection of the peak-locked averages supports this, with several participants showing clear harmonics at 60 Hz, rather than 40 Hz (for examples, Fig. S8A). Moreover, we observed a negative correlation between harmonic power at 40 and 60 Hz across participants (*r* = −0.677, *P* < 0.001; see Fig. S8B), suggesting participants show responses at either 40 or 60 Hz but not both. These results suggest that harmonic responses to 20 Hz stimulation are influenced by the resonant frequency of endogenous gamma oscillations.

It is worth noting that the analyses in this subsection were between-participant and conducted with a relatively small sample (experiment 3: *n* = 13), which likely overestimates the effect size. Therefore, it will be of interest to see how these results scale in larger datasets. Nonetheless, these observations suggest that harmonic responses to rhythmic stimulation depend on the participant-specific resonant frequency of endogenous alpha/gamma oscillations.

### Co-occurring harmonic and fundamental neural responses separably influence human visual perception

Finally, we asked whether harmonic responses to rhythmic stimulation impact human behavior by investigating whether the phase of harmonic alpha-band oscillations impacts visual perception (Busch et al. 2009; Mathewson et al. 2009; Dugué et al. 2011; Griffiths et al. 2022; Fakche and Dugué 2024).

**Figure 8:**
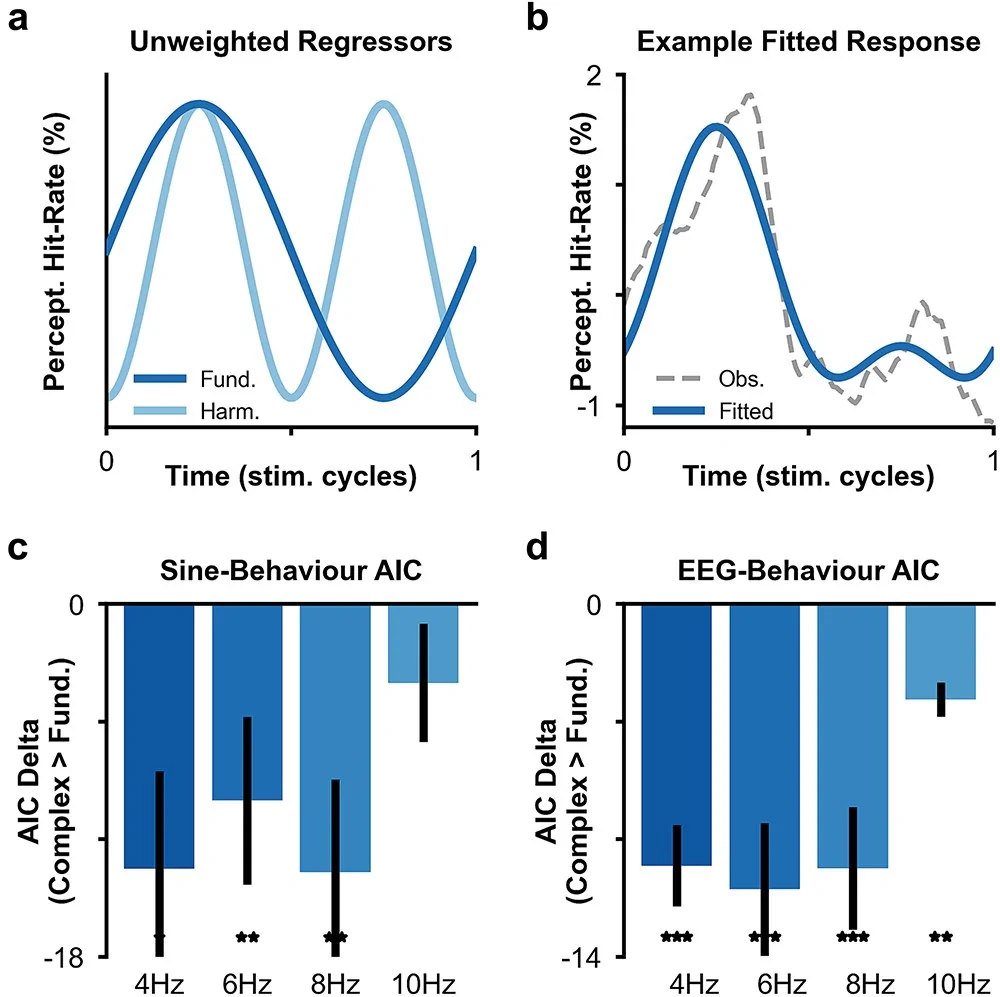
Fundamental and harmonic neural responses interact to impact visual perception. a) We modeled perceptual hit rate (ie the behavioral responses) over time as the weighted sum of 2 sine waves—1 at the fundamental frequency (dark blue) and 1 at the second harmonic (light blue). b) Example fit to behavioral data. Dotted gray line indicates perceptual hit rate over the course of a stimulation cycle. Solid blue line indicates the fitted sine waves. c) To identify whether how the 2 sine waves impacted modeling fitting, we compared the AIC for a simple model using only the sine wave at the fundamental frequency against a complex model using both sine waves. Aside from 10 Hz, we find a significant improvement in model fit when using the complex model. Bars indicate mean AIC across participants, error bars indicate standard error in AIC across participants. d) Example model fit using IMF waveshapes in place of sine waves. When fitting the models using IMF waveshapes in place of sine waves, we replicate our results, indicating that neural responses to stimulation at fundamental and harmonic frequencies interact to modulate perceptual performance. **P < 0.01; *P < 0.05.

To address this, we expanded on the original analysis of experiment 3, which demonstrated that visual perception fluctuates according to the phase of the fundamental frequency of stimulation (Fakche and Dugué 2024). We binned behavioral data according to the phase of rhythmic light stimulation and then used sine waves to model this behavioral time series. We built 2 models: a “simple” model consisting of a single sine wave at the fundamental frequency and a “complex” model consisting of 2 sine waves, one at the fundamental frequency and the other at the second harmonic (see Fig. 8a and b).

We used the AIC as the primary metric for comparing the 2 models (see Methods for justification; the BIC is reported alongside for completeness). For both measures, a decrease in information criterion for the complex model means that the harmonic sine wave improves the fit to behavioral data relative to what the fundamental sine wave achieved in isolation (after accounting for the difference in the number of free parameters). Indeed, we found that the “complex” model explained behavioral performance to a significantly greater degree than the “simple” model for all stimulation conditions except 10 Hz (ie the one condition that did not produce a harmonic response; 4 Hz: *z* = −5.24, *P* = 0.019; 6 Hz: *z* = −5.15, *P* = 0.006; 8 Hz: *z* = −5.78, *P* = 0.004; 10 Hz: *z* = −9.21, *P* = 0.063; see Fig. 8c). The BIC produced a similar pattern, though its more conservative complexity penalty rendered the 6 Hz condition non-significant (BIC; 4 Hz: *z* = −4.56, *P* = 0.046; 6 Hz: *z* = −4.15, *P* = 0.155; 8 Hz: *z* = −4.89, *P* = 0.035; 10 Hz: *z* = −5.05, *P* = 0.960). To rule out the possibility that the AIC did not sufficiently penalize the “complex” model, we ran a control analysis using phase-shifted versions of the harmonic IMF, finding that the true “complex” model better explained behavioral changes in performance than any other phase-shifted “complex” model (see Fig. S4). These results suggest that the phase of harmonic responses impacts visual perceptual performance in a way that is complementary, but separable, to responses at the fundamental frequency.

To tie this back to the putative alpha oscillations in the EEG, we refined the above analysis by replacing the sine waves with participant-specific waveshapes (extracted from the fundamental and harmonic IMFs). This approach more precisely captures each participant’s cortical alpha activity than an idealized sinusoid, and should therefore provide a more sensitive test of whether harmonic neural responses contribute to behavior. Indeed, we found that the “complex” model explained behavioral performance to a significantly greater degree than the “simple” model (AIC: 4 Hz: *z* = −21.24, *P* < 0.001; 6 Hz: *z* = −17.22, *P* < 0.001; 8 Hz: *z* = −19.62, *P* < 0.001; 10 Hz: *z* = −4.65, *P* = 0.004; see Fig. 8d; BIC: 4 Hz: *z* = −14.76, *P* = 0.002; 6 Hz: *z* = −6.62, *P* = 0.009; 8 Hz: *z* = −3.553, *P* = 0.014; 10 Hz: *z* = −0.052, *P* = 0.292; note that the 6 Hz effect is significant here under BIC, suggesting that the earlier null effect reflects limited sensitivity of the idealized sine-wave model rather than an absence of a harmonic contribution to behavior).

To assess whether fundamental and harmonic contributions to behavior are separable, we correlated the betas for the fundamental and harmonic regressors across participants, for each stimulation condition. No significant correlation was observed for any condition in either the sine-wave model (4 Hz: *r* = −0.17, *P* = 0.586, BF₁₀ = 0.239; 6 Hz: *r* = 0.17, *P* = 0.581, BF₁₀ = 0.564; 8 Hz: *r* = 0.06, *P* = 0.839, BF₁₀ = 0.400; 10 Hz: *r* = −0.41, *P* = 0.168, BF₁₀ = 0.161) or the waveshape model (4 Hz: *r* = −0.35, *P* = 0.238, BF₁₀ = 0.175; 6 Hz: *r* = 0.35, *P* = 0.245, BF₁₀ = 0.704; 8 Hz: *r* = 0.01, *P* = 0.970, BF₁₀ = 0.351; 10 Hz: *r* = −0.35, *P* = 0.239, BF₁₀ = 0.175). Bayes factors were generally consistent with the null hypothesis, though evidence strength ranged from anecdotal to moderate (Van Doorn et al. 2021), likely reflecting the small sample size. Taken together, these results reveal no evidence to suggest the fundamental and harmonic influences over perception co-vary across participants, consistent with the view that they reflect separable contributions to behavior.

These results indicate that harmonic alpha activity induced by theta-band stimulation can impact near-threshold visual perception. This harmonic effect is summed with the effect from neural responses at the fundamental frequency, indicating that fundamental theta-band and harmonic alpha-band responses to stimulation have separable causal effects on behavioral performance.

## Discussion

We demonstrate that rhythmic light stimulation elicits multiple concurrent neural responses that have separable impacts on human perception. Using EMD (Huang et al. 1998; Quinn et al. 2021) to distinguish oscillations from artifacts of a single non-sinusoidal waveform (Aru et al. 2014; Cole et al. 2017; Cole and Voytek 2017; Schaworonkow 2023), we show that rhythmic stimulation elicits harmonic responses within the canonical gamma and alpha bands in the visual cortex. These responses influence human perception in a manner distinct from co-occurring responses at the fundamental frequency, with gamma-band responses contributing to oscillatory coding (Lisman and Jensen 2013; Fries 2015; Griffiths and Jensen 2023) and harmonic alpha responses contributing to phase-dependent visual perception (Busch et al. 2009; Mathewson et al. 2009; Dugué et al. 2011; Griffiths et al. 2022; Fakche and Dugué 2024; see also Griffiths et al. 2023, for how harmonic gamma-band responses impact associative memory). We observe these effects across 3 independent datasets, 2 distinct tasks, 2 recording modalities, and multiple stimulation frequencies, using a variety of preprocessing pipelines, stimulation parameters, stimulation hardware (ie monitors/projectors), and baseline conditions. This steps beyond past work that has demonstrated the presence of FFT-derived harmonics in electrophysiology (Fawcett et al. 2004; Pastor et al. 2007; Kim et al. 2011; Heinrichs-Graham and Wilson 2012; Gulbinaite et al. 2019; Retter et al. 2021) by revealing the nature of these harmonics: namely, that these reflect concurrent oscillatory responses, rather than simply reflect harmonic components of a single, non-sinusoidal oscillation. We propose that harmonic oscillatory responses to rhythmic light stimulation are a reliable and pervasive phenomenon that influences human perception in ways unique to neural responses at the fundamental frequency. More generally, we suggest that oscillatory responses to rhythmic brain stimulation are more complex than is often acknowledged, and accounting for this complexity may be key to advancing our understanding of how rhythmic brain stimulation impacts cognition and behavior.

### “Neural side effects” in neuromodulation

Most rhythmic stimulation experiments seek to match stimulation frequency to a specific oscillatory target. We demonstrate that this approach may unintentionally modulate other oscillations that have a harmonic relationship with the stimulation frequency. Therefore, these harmonic effects (and their behavioral consequences) could be considered “neural side effects.” This provides a possible explanation of variability in rhythmic stimulation studies: unaccounted neural responses introduce a series of frequency- and participant-specific confounds that bias results and interpretations. Accounting for these “side effects,” therefore, may enhance the accuracy and precision of studies investigating how rhythmic stimulation influences human perception, cognition, and behavior, leading to more conclusive evidence for causal mechanisms and improved efficacy of neural interventions.

We envisage that these effects could be accounted for analytically and/or methodologically. First, it can be handled analytically, as we have done above: decomposing the recorded MEG/EEG signal into IMFs and incorporating them into a single model that aims to explain behavior (see Fig. 8). This modeling approach means the IMFs cannot explain the same variance in behavior, and therefore helps dissociate their contributions to changes in behavior. On a therapeutic level, however, it may be better to tackle this issue methodologically, minimizing harmonic neural responses to stimulation by modifying the stimulation protocol itself. Empirical demonstrations of such methods are uncommon, but modeling work suggests that adding white noise to the stimulation period may be able to suppress neural responses beyond the fundamental frequency (Duchet et al. 2023). Notably, however, suppressing the harmonic response may undermine an intervention if the harmonic response is the sole driver of the observed cognitive change (eg gamma oscillatory coding; Fig. 5). Therefore, one needs to take careful consideration of the strategy used to account for the harmonics so that they can maximize interpretability and impact without abolishing the effect of interest.

### Entrainment, resonance, or steady-state visually-evoked responses?

We show that fundamental and harmonic responses to rhythmic stimulation differ in spectral, spatial, and temporal dynamics. But do they also differ in how they are generated?

There is an ongoing debate about the neural mechanisms that generate electrophysiological responses to rhythmic stimulation (Capilla et al. 2011; Herrmann et al. 2016; Zoefel et al. 2018; Duecker et al. 2021). An electrophysiological signature of rhythmic stimulation can arise when (a) an endogenous oscillator adjusts its frequency and phase to match the input (“entrainment”; Lakatos et al. 2019); (b) an endogenous oscillating response resets with each stimulation pulse (“resonance”), or (c) aperiodic steady-state visually-evoked potentials (“SSVEPs”) are superimposed (Capilla et al. 2011). Distinguishing between these mechanisms is crucial for both fundamental and translational research as the underlying mechanism dictates how rhythmic stimulation impacts cognition. For example, Alzheimer’s disease is associated with disrupted gamma oscillations (Mably and Colgin 2018), but if gamma-band stimulation only elicits SSVEPs (rather than modulating dysfunctional gamma oscillations), the intervention would be unlikely to tackle the underlying cause of the problem.

While it remains ambiguous whether a rhythmic response at the fundamental frequency can reflect entrainment (Capilla et al. 2011; Herrmann et al. 2016; Zoefel et al. 2018; Duecker et al. 2021), we suggest that the harmonic responses observed here are most parsimoniously accounted for by entrainment because:

1) The harmonic response frequency is always an integer multiple of the fundamental frequency (that is, the frequency of the harmonic response shifts with the input frequency, as in entrainment). Resonance, in contrast, would predict that the higher frequency response would have a fixed numerical value matching the resonant frequency of the endogenous rhythm.
2) There are 2 cycles of the harmonic response per stimulation pulse, making it difficult to attribute this to SSVEPs. One could argue that harmonics reflect a reversal (Norcia et al. 2015; Blanpain et al. 2024), with SSVEPs occurring at both pulse onset and offset, but experiments 1 and 2 use asymmetric stimulation protocols (Fig. 2b), so reversals cannot explain the equidistant peaks observed in peak-locked averages of these experiments (Fig. 3a). Moreover, reversals cannot explain why some participants showed responses at the third harmonic (Fig. S8).
3) The harmonic responses are strongest when their frequencies align with endogenous resonant frequencies (eg the Arnold Tongue-like pattern in harmonic responses in experiment 1), and vary across participants based on individual differences in resting oscillatory frequency. This dependence on endogenous oscillatory alignment is a characteristic of entrainment, but not of SSVEPs nor resonance.

Therefore, we hypothesize that harmonic responses reflect entrained oscillations. We acknowledge that the present data do not definitively establish this interpretation. Whether rhythmic stimulation entrains endogenous oscillations, even at the fundamental frequency, has been debated for decades (Capilla et al. 2011; Herrmann et al. 2016; Zoefel et al. 2018; Duecker et al. 2021), and resolving this question has required, and will continue to require, converging evidence across multiple studies and methodologies. The present data provide the empirical basis for this investigation and generate a number of testable predictions. For example, a confirmatory test could involve investigating the persistence of oscillatory responses after the offset of stimulation (VanRullen and Macdonald 2012; Hanslmayr et al. 2014; Duecker et al. 2024) (ie “oscillatory echoes”): if the oscillatory responses persist at the exact harmonic frequency, it would provide strong evidence for entrainment. If, in contrast, oscillatory responses either immediately shift to a neighboring harmonic frequency or disappear entirely, responses might better be ascribed to resonance or steady-state evoked responses. Unfortunately, in all our experiments, the offset of the stimulation coincided with the onset/change of other facets of the task (eg the onset of a response prompt), introducing a source of covariance that precludes the testing of the hypothesis here.

The responses at the fundamental that we observe here present the same interpretative challenges as previous work (Capilla et al. 2011; Herrmann et al. 2016; Zoefel et al. 2018). Since fundamental responses appear across all stimulation frequencies, SSVEPs likely contribute significantly to their generation. This hypothesis is supported by the observation that fundamental responses diminish in power when harmonically responding alpha activity increases, mirroring reductions in early visually evoked responses during periods of high alpha activity (Iemi et al. 2019). However, this does not rule out the possibility of entrainment contributing to the fundamental response (eg Notbohm et al. 2016). Indeed, it is entirely possible that the macroscopic neural responses to stimulation we record here reflect the summation of SSVEP and entrained responses (see Fig. S9). As our approach separates neural responses by frequency, it cannot disentangle multiple processes occurring at the same frequency, meaning the SSVEP versus entrainment debate for responses at the fundamental frequency remains an open question for future research.

In sum, harmonic responses most parsimoniously align with the concept of entrainment, whereas fundamental responses remain ambiguous. As the arguments linking harmonic responses to entrainment face fewer confounds than those at the fundamental, we propose that future entrainment research may benefit from focusing on harmonic responses, which offer a way to circumvent pervasive questions about the mechanisms underlying rhythmic electrophysiological responses, and which may ultimately enable us to arrive at stronger and clearer conclusions about how neural oscillations causally impact perception, cognition, and behavior.

### Causal role of alpha phase in visual perception

There is considerable interest in the role of alpha oscillations in visual perception. Many correlative studies suggest that perceptual behavior depends on the phase of alpha (Busch et al. 2009; Mathewson et al. 2009; Dugué et al. 2011; Griffiths et al. 2022; Fakche and Dugué 2024), but results are not always consistent (Ruzzoli et al. 2019; Zazio et al. 2021) with evidence indicating that these effects may be mediated by other phenomena (Keitel et al. 2022; eg oscillatory power [Mathewson et al. 2009; Fakche et al. 2021]; cross-region interactions [Griffiths et al. 2022]). Causal studies have aimed to establish the primacy of visual alpha oscillations by directly manipulating these rhythms to modulate behavior (Mathewson et al. 2012; de Graaf et al. 2013; Jaegle and Ro 2014; Spaak et al. 2014; Kizuk and Mathewson 2017; Fiene et al. 2022; Fakche and Dugué 2024). While such approaches do modulate behavior, these studies are susceptible to confounds such as phase-dependent phosphenes or luminance changes that impact vision. This leaves the possibility that stimulation-induced changes in neural activity reflect SSVEPs rather than modulations of true oscillations. Harmonic responses to stimulation, however, circumvent these concerns and, therefore, may be key to resolving this debate.

We show that fundamental and harmonic responses to theta-band stimulation have complementary influences on visual perceptual behavior. As the fundamental and harmonic phases are orthogonal, luminance differences at the fundamental frequency cannot explain the harmonic effect. Moreover, as discussed above, the harmonic response cannot be attributed to an SSVEP. Therefore, the contributions of the harmonic effect are best ascribed to modulations of endogenous alpha oscillations, supporting the case for a causal role of alpha oscillatory phase in visual perception.

As stimulation was delivered continuously across a block, and there was no block without stimulation to act as a baseline, we were unable to assess whether these harmonic responses also impacted links between prestimulus alpha power and other aspects of perception (eg Benwell et al. 2017, 2021; Samaha et al. 2017), but minor modifications to our approach would allow researchers to investigate this, providing insight into the causal role of alpha power on perception and how its interacts with alpha phase-dependent perceptual effects (Mathewson et al. 2009).

### Gamma oscillations and oscillatory coding

Incoming visual sensory information is thought to be represented in a gamma oscillatory code (Thut et al. 2012; Lisman and Jensen 2013; Bieri et al. 2014; Fries 2015; Watrous et al. 2015; Han et al. 2021). We find support for this idea in experiment 2, where we observed that representational fidelity of stimulus-specific visual information rhythmically fluctuated in accordance with the gamma-band harmonic responses to 34 Hz stimulation. Notably, the fundamental response showed no effect, indicating a dissociation between fundamental and harmonic responses in the representation of sensory information. These results further support the theory that harmonic responses reflect changes in true oscillatory activity which contribute to neural processing distinct from responses at the fundamental.

The detection of gamma-band oscillatory codes is challenging to test in humans as (i) gamma frequencies overlap with muscle artifacts (Jerbi et al. 2009; Muthukumaraswamy 2013), introducing noise into measurement, (ii) there is substantial variability in gamma frequencies across participants (Griffiths et al. 2019, 2021; Duecker et al. 2021), complicating group-level analyses, and (iii) stimulus properties impact the exact frequency of the gamma response (Hermes et al. 2015b), introducing across-trial variability. We show that rhythmic light stimulation can circumvent these issues by amplifying the code at a known frequency, both across participants and across naturalistic visual stimuli. This offers a new means to investigate the causal role of oscillatory coding across cognition and behavior (eg the processing of complex visual scenes [Bonnefond et al. 2024]; theta-gamma coded trajectories in spatial navigation [Lisman and Jensen 2013; Colgin 2016]; cross-regional communication during memory formation and retrieval [Colgin et al. 2009; Griffiths et al. 2019; Pacheco Estefan et al. 2019; Griffiths and Jensen 2023]) that may otherwise have proven impossible to assess.

### Alternative explanations

Harmonic responses to stimulation are often viewed as artifacts (Aru et al. 2014; Norcia et al. 2015; Cole et al. 2017; Schaworonkow 2023). However, we believe that oft-cited reasons for doing so, reviewed in turn below, have no bearing on our findings:

- Fourier-related artifacts: Fourier-derived power spectra of non-sinusoidal oscillations produce harmonics at multiple frequencies (Aru et al. 2014; Cole et al. 2017; Cole and Voytek 2017; Schaworonkow 2023), so harmonic effects observed using this approach are often assumed to be artifacts. We address this issue by using EMD (Huang et al. 1998; Quinn et al. 2021), to distinguish putative harmonic components from non-sinusoidal fundamental responses (Fabus et al. 2021) and then test whether these separated components exhibit distinct neural and behavioral properties. Critically, we show that harmonic responses to stimulation act in differing ways from fundamental responses on both a neural and behavioral level—a result that would not occur if the harmonic response were a reflection of a non-sinusoidal response at the fundamental. Further evidence to suggest that our effects do not reflect non-sinusoidal artifacts includes (a) the observation of a singular harmonic response (typically at the second harmonic, but see Fig. S3) which differs from non-sinusoidal artifacts (which produce responses at multiple harmonic frequencies [Norcia et al. 2015]) and (b) the observation that harmonic effects were strongest when they approximate endogenous oscillatory frequencies, which differ from non-sinusoidal artifacts which produce harmonics regardless of stimulation frequency (Herrmann 2001).
- Stimulation reversals: Reversals occur when the brain elicits evoked responses to both the onset and offset of the stimulation pulse. If the “on” and “off” durations match, second harmonics can be attributed to reversals (eg Norcia et al. 2015; Blanpain et al. 2024). However, this was not the case in experiments 1 and 2, where the pulse was only presented for a single frame (8.33 ms in experiment 1; 2.08 ms in experiment 2; see Fig. 2b), meaning reversals are unlikely to explain these effects. Moreover, reversals fail to account for participants showing harmonic responses at the third harmonic (ie 3 oscillatory cycles per pulse; see Fig. 5e) or why harmonic responses are strongest when they align with endogenous oscillations. More generally, the high frequency of these reversals (ie 120 Hz in experiment 1; 480 Hz in experiment 2) means they will likely be decimated by the low-pass filtering properties of the thalamus (Hawken et al. 1996; Duecker et al. 2024). This means any reversal effect, or more general “on–off” effect relating to stimulation waveshapes (Norcia et al. 2015), would not reach the occipital sources of our main effects.
- Filtering artifacts: Narrowband filtering can distort stimulation-evoked responses to create spurious oscillations (Helfrich et al. 2019). We mitigated this by using peak-locked averages in place of narrowband filters. Furthermore, filtering artifacts cannot explain why, when using the same filters for all data, we see inter-subject and frequency-dependent variability in harmonic responses to stimulation.
- Dipoles: Second harmonics could be explained by the EEG picking up mirrored activity from dipoles (that is, the fundamental and harmonic responses are 2 sides of the same coin). However, this cannot explain (i) why the fundamental and harmonic responses have distinct effects on visual perception and oscillatory coding; (ii) why the fundamental and harmonic responses are uncorrelated over time in experiments 1 and 2; and (iii) why some participants show harmonic responses at the third harmonic (see Fig. S3).

Therefore, our results indicate that the effects reported here are genuine harmonic responses to stimulation, distinct from those at the fundamental response, which should not be indiscriminately dismissed as artifacts.

### Future directions

Our findings question the idea of a 1-to-1 mapping between rhythmic stimulation and neural response. Instead, they suggest that rhythmic input elicits a multitude of neural responses, but much remains to be explored about these concurrent responses, including:

- The impact of harmonic responses on cognition: Perhaps the most important future direction is ascertaining whether harmonic responses to rhythmic light stimulation have a meaningful impact on behavior. Indeed, a primary motivator for using rhythmic light stimulation in neuroscientific research is to gain a causal understanding of how neural dynamics shape behavior, and whether these insights can be translated into therapies for neuropsychological illnesses. Our observation of an effect of harmonic oscillatory responses on near-threshold visual perception suggests that harmonic responses could, more generally, impact behavior (a proposal supported by other work demonstrating improvements in episodic memory retrieval linked to harmonic responses to rhythmic stimulation [Griffiths et al. 2023]). However, given the many cognitive domains which can be influenced by rhythmic light stimulation, including attention (Gulbinaite et al. 2017; Wiesman and Wilson 2019), multisensory integration (Gomez-Ramirez et al. 2011), working memory (Albouy et al. 2022; Pileckyte and Soto-Faraco 2024) and long-term memory (Williams 2001; Clouter et al. 2017; Roberts et al. 2018), and the neuropsychological disorders which stand to benefit from rhythmic light stimulation therapies (eg Alzheimer’s disease; Adaikkan et al. 2019; Martorell et al. 2019; Chan et al. 2022), extensive further work is required to determine whether harmonic oscillatory influences on behavior are the rule, or whether the effects we observe here are unique to perception.
- Data-driven detection of neural responses: We adopted a hypothesis-driven approach to defining the neural responses within our transfer functions, but what if this is not feasible? In such instances, data-driven approaches such as generalized eigendecomposition (GED) or bicoherence may offer solutions. For example, GED can isolate a principal neural response to rhythmic stimulation (Cohen and Gulbinaite 2017) and separate harmonic components of an FFT (Mora-Cortes et al. 2018; Gulbinaite et al. 2019). Bicoherence, meanwhile, could identify candidate harmonic frequencies that are phase-coupled to the fundamental stimulation frequency (Dumermuth et al. 1971; Sheremet and Qin 2025). Integrating these approaches with EMD may enable data-driven ways to capture multiple, separable oscillatory responses to rhythmic stimulation.
- Spatial origins: Our topographic analyses revealed that the fundamental and harmonic responses can be statistically separated, but it is an open question whether these statistically-separable sources are also physically separable (ie arising in 2 separate brain regions), partially overlapping, or whether one response emerges from a subspace of the other response (as suggested by the topographies in experiment 2). Understanding this may be key to developing spatially-targeted stimulation protocols that seek to modulate only 1 response (or suppress the other response).
- Causal chains: Our analysis assumed that the fundamental and harmonic responses to rhythmic stimulation occur independently. However, it remains possible that 1 neural response impacts another. Such interactions are hinted at by the negative correlation we observe between harmonic alpha power and fundamental theta responses, and by other studies which stimulate 1 region with the aim of having knock-on effects in task-critical regions (Wang et al. 2014; Inman et al. 2018; Warren et al. 2019). Accounting for these interactions would further clarify the causal roles of different neural responses in cognition.
- Subharmonics: We focused on harmonic neural responses to rhythmic stimulation as this is well-documented in physics (Pikovsky et al. 2001) and biology (Guevara et al. 1988; Crevier and Meister 1998; Peylo et al. 2022; Jiménez et al. 2024), but could this principle generalize to subharmonics? Previous studies cast a light on this, showing that 130 Hz stimulation of the basal ganglia modulates 65 Hz gamma activity (Swann et al. 2016; Sermon et al. 2023, 2024). Open questions remain about whether this utilizes the same mechanisms for generation as harmonic responses. Nonetheless, these results add further support to our claim that a single rhythmic stimulation protocol can elicit a complex array of neural responses.
- Generalizability to TMS/transcranial alternating current stimulation (tACS): We postulate that TMS and tACS will produce similar effects to what we observe with rhythmic light stimulation. Indeed, theta-band tACS elicits alpha-band harmonics in ferrets (Ali et al. 2013). Moreover, as responses to rhythmic light stimulation are likely weaker than those elicited by TMS/tACS, and as harmonic responses to rhythmic inputs require a strong input (Pikovsky et al. 2001), one might hypothesize that harmonic responses may be more pronounced in TMS/tACS. In other words, our effects may be understating rather than overstating the influence of harmonic responses on neural and behavioral outcomes.
- Precision and personalized neurostimulation: Accounting for complex neural responses in future research and clinical designs can improve the reliability of scientific findings and efficacy of clinical treatment. For example, developing stimulation protocols that maximize the target neural response while minimize other neural “side-effects” (eg Duchet et al. 2023), or including additional control conditions (eg harmonic frequencies) could provide greater insight into the mechanisms underpinning neural and behavioral changes.

### Summary

Rhythmic light stimulation (and neuromodulation more broadly) plays a critical role in translating fundamental neuroscience into clinical applications. However, the results of such studies are often highly variable, limiting the development of effective treatments for cognitive and neurological disorders. Here, we show that neural responses to rhythmic light stimulation are more complex than is often acknowledged. Specifically, rhythmic light stimulation elicits multiple neural responses with separable spectral, spatial, and temporal profiles, each exerting separable influences over human perception and varying across individuals. These findings open new avenues of research, including asking whether harmonic responses to rhythmic light stimulation exert similar impacts on other cognitive domains, and whether harmonic responses can enhance the efficacy of rhythmic light stimulation in neurotherapies (Adaikkan et al. 2019; Martorell et al. 2019; Chan et al. 2022; Soula et al. 2023). Indeed, a deeper understanding of this phenomenon may well reshape how we approach neuromodulation in the lab, in the clinic, and beyond.

## Supplementary Material

supplementary_material_bhag152
