# Supplementary material for: Rhythmic stimulation elicits multiple, concurrent neural responses
Source: Cereb Cortex. 2026 Sep 22;36(9):bhag152. doi: 10.1093/cercor/bhag152 (PMC13596740; doi:10.1093/cercor/bhag152)
Supplement: Supplementary file 1 [file supplementary_material_bhag152.docx]

**Supplementary Material**

for

***Rhythmic stimulation elicits multiple, concurrent neural responses***

Benjamin J. Griffiths^1,2^, Katharina Duecker^3^, Camille Fakche^4^, Laura Dugué^5,6^, Andrew Quinn^1^, & Ole Jensen^1,7,8^

1. Centre for Human Brain Health, University of Birmingham, UK
2. School of Psychology, University of Nottingham, UK
3. Department of Neuroscience, Brown University, USA
4. Centre de Recherche en Neuroscience de Lyon, France
5. Université Paris Cité, CNRS, Integrative Neuroscience and Cognition Center, F-75006 Paris, France
6. Institut Universitaire de France (IUF), Paris, France
7. Oxford Centre for Human Brain Activity, University of Oxford, UK
8. Department of Experimental Psychology, University of Oxford, UK

**Figure S1. FFT mistakes non-sinusoidal waveforms for multiple oscillations.** Past studies have found spatial separation between harmonic components of neural responses to rhythmic stimulation when using the FFT. Some may view this as evidence for distinct oscillatory responses to stimulation, but this can reflect small changes in waveshape at the fundamental. To demonstrate this, we simulated two non-sinusoidal 10Hz oscillations (left column; black lines) and added pink noise (grey lines) [for the sake of this hypothetical, consider these different waveforms arose in different spatial locations: V1 vs. V2]. The “V2” waveshape deviates more from a pure sine wave than the “V1” waveshape, so it can be considered more non-sinusoidal. We applied FFT (middle column) and EMD (right column) to both. The FFT produced a small but perceptible harmonic at 20Hz for the “V2” waveshape that was driven by the non-sinusoidal waveform. EMD produced no such harmonic. When directly contrasting the power spectra of the two waveforms (bottom row), the FFT showed greater 10Hz power for “V1” and greater 20Hz power for “V2” (mimicking earlier results; Fawcett et al., 2004; Gulbinaite et al., 2019a; Heinrichs-Graham & Wilson, 2012; Kim et al., 2011; Pastor et al., 2007a; Retter et al., 2021). This suggests that spatial separation methods applied to FFT power spectra could lead to the misinterpretation of differences in waveshape as the presence of a harmonic oscillation. Contrasts of the EMD spectra found no harmonic effect (bottom right), suggesting EMD-based analyses are not susceptible to this problem. Note that while the EMD analyses introduce some quirks of their own (namely that the more non-sinusoidal “V2” waveshape produces a power spectrum with a wider distribution around 10Hz than the “V1” waveshape), this does not impact effects at the harmonic: the key focus of the questions of this manuscript.

**Figure S2. The impact of using an iterated masked sift approach to EMD and various forms of neural response to rhythmic stimulation at 24Hz (second harmonic = 48Hz).** Columns depict critical stages of the pipeline used in the main text; rows reflects different neural responses. **(A)** We simulated two independent oscillations at *f* and *2f* (first column; our hypothesis), which were locked to a stimulation pulse (dotted grey line). We summed these oscillations together with pink noise to create a summed signal emulating what might be recorded by an MEG sensor or EEG channel (second column). We ran peak-locked averaging (as described in the main text) to accentuate rhythmic features and suppress aperiodic noise (third column), followed by an iterated masked sift and were able to separate the two summated oscillations from each other and the pink noise introduced in the summed signal. Altogether, this demonstrated that an iterated masked sift for EMD can recover multiple oscillations from a single, noisy recording. **(B)** We applied the same logic to a single, non-sinusoidal oscillation at *f*Hz. This emulates cases of non-sinusoidal oscillations phase-locked to stimulation and superimposed steady-state event-related potentials (SSVEPs). When running our pipeline on this signal, no harmonic component was identified, suggesting our approach is not likely to mislabel a non-sinusoidal waveshape as two separate oscillations at *f*Hz and *2f*Hz (see also Supplementary Figure 1). **(C)** We simulated multiple non-sinusoidal responses at *f*Hz to emulate a scenario in which rhythmic stimulation evokes multiple event-related potentials with different time delays (e.g., N1, P1, and N2). The time delays between the evoked components reduces the clarity of the SSVEP in the summed signal and peak-locked average, but the iterated masked sift is still able to extract the rhythm at *f*Hz. As in Panel B, these non-sinusoidal responses do not produce effects at *2f*. **(D)** Finally, we consider a scenario in which rhythmic stimulation elicits a non-sinusoidal response at *f* and a (sinusoidal) harmonic response at *2f*. As in Panel A, the harmonic response can be separated from the fundamental response. This suggests that the harmonic response can be recovered even when the fundamental response is atypical.

**Figure S3. Mean IMF frequency as a function of IMF number.** The EMD implementation used here resolves progressively slower IMFs. The dotted line indicates the slowest stimulation frequency in each experiment. Five IMFs were consistently sufficient to resolve all stimulation frequencies used across experiments. This includes an final IMF which separates the slowest stimulation frequency from lower-frequency activity (e.g., slow drifts). Bars show mean IMF frequency across participants; error bars show standard error of the mean.

**Figure S4. Change in AIC when phase-shifting the harmonic IMF.** While AIC does penalise models for increased complexity (theoretically accounting for the difference in regressors between the fundamental and complex models presented in the main text), this penalisation is not always sufficient. Therefore, to complement the analysis presented in the main text, we ran a control analysis where we computed the complex model AIC after phase-shifting the harmonic IMF systematically from zero to 2π radians. If the improvement we saw in model fit for the complex relative to the fundamental model was simply due to the addition of an additional regressor, then AIC should be consistent across all phase shifts of the harmonic IMF. If, however, the observed harmonic IMF is explaining additional variance in the data above and beyond the fundamental model, then the improvement in model fit (i.e., when AIC is lowest) should be greatest for the harmonic IMF without phase-shifting. Indeed, this is exactly what we observed in the polar plot above. In this plot, the dark blue line represents normalised AIC, where values below zero (the black dotted line) indicate a better model fit than the average model fit for all phase shifts. When there is no phase shift (i.e., angle = 0), model performance is significantly greater than the mean AIC across phase shifts (z = -4.77, p = 0.037), indicating that the non-phase-shifted data explains more variability in the behavioural data than would be expected from simply adding any harmonically oscillating regressor. These results support those in the main text and suggest that harmonic alpha activity induced by theta-band stimulation can influence near-threshold visual perception.

**Figure S5. The iterated masked sift (itEMD) reliably separates a fundamental and its harmonic across a broad range of mask amplitudes, without spuriously splitting a pure oscillation.** Mask amplitude is a free parameter in itEMD. To reduce experimenter degrees of freedom, we used the toolbox default (amplitude = one standard deviation of the previous IMF) and use the simulations presented here to verify that the default is a suitable choice for our analysis. To this end, we simulated 2s signals (sample rate: 1000Hz) comprising a 10Hz fundamental and its 20Hz harmonic summed with pink (1/f) noise, with the amplitude of the harmonic varying as a ratio of the amplitude of the fundamental. **(A)** The resulting noiseless complex waveshape for four harmonic-to-fundamental amplitude ratios (2f/f). As the ratio increases the waveform becomes progressively more peaked and asymmetric, with the harmonic contributing a secondary bump at the fundamental's trough. Though pink noise was included in the simulations, it is removed from this panel to help visualise how changes in the harmonic-to-fundamental ratio impacts waveshape. **(B)** Probability of successful separation (i.e., the percentage of simulations which recovered two distinct IMFs whose mean instantaneous frequencies fall within ±20% of f and 2f, respectively) as a function of harmonic amplitude ratio (y-axis) and itEMD mask amplitude (x-axis). Separation is reliable, but can fail at extremes when the harmonic is too small to be resolved from the fundamental, or when the mask amplitude is large relative to a moderate harmonic, with the minimum ratio required for separation rises with mask amplitude. The default mask amplitude sits at the centre of the reliable region. **(C)** Probability of a false-positive split (i.e., the percentage of simulations in which a single 10 Hz sinusoid [plus pink noise, no harmonic] was erroneously decomposed into distinct f and 2f IMFs) as a function of mask amplitude. This remains at ~0 across the full range of tested mask amplitudes, indicating that itEMD does not produce spurious harmonics from a single, noisy oscillation.

**Figure S6.** **Robustness of the spectral-separation results to the itEMD mask-amplitude parameter.** The spectral-separation analysis (Figure 3) decomposes peak-locked responses using iterated-mask empirical mode decomposition (itEMD) and tests, for each condition, whether spectral power at the driven fundamental frequency (f) and its first harmonic (2f) exceeds baseline. We investigated whether changes in mask amplitude would impact the statistical conclusion drawn from a given analysis (i.e., “was the effect significant?”). Each panel is a heatmap of the cluster-level z statistic for the stimulation-versus-baseline contrast (paired-samples, cluster-based permutation test), for one experiment and one spectral component. Rows correspond to the three experiments. The left column shows the fundamental (f) component and the right column the harmonic (2f) component. Within each panel, the x-axis is the itEMD mask amplitude (0.2, 0.5, 1, 2, 5; the default of 1 is shown in bold and outlined in black), and the y-axis is the power at the response frequency tested in that experiment. Cell colour depicts the cluster z value. Cluster significance is overlaid on every cell (*** p ≤ .001, ** p ≤ .01, * p ≤ .05, ^x^ p ≤ .1). Across every experiment the fundamental response remains significant over the full range of mask amplitudes, with a single exception at 30 Hz under the most extreme setting (mask amplitude = 5) in Experiment 1. The harmonic responses that are significant at the default largely retain significance across the range. The spectral-separation results are therefore not driven by the choice of mask amplitude.

**Figure S7. Across-participant correlations relating absolute distance between IAF and harmonic frequencies to spectral power at the harmonic frequency. Dots reflect individual participants; the coloured line reflects the line of best fit. The 6Hz and 8Hz stimulation harmonic responses to stimulation decreasing in amplitude as the stimulation frequency strays further from the individual alpha frequency.**

**Figure S8. Across-participant variability in harmonic gamma-band responses. (A)** Peak-lock averages for several participants when receiving 20Hz light stimulation. Some participants show harmonic responses at 40Hz (left; also see Figure 3A), others respond at 60Hz (middle) or display dynamics reminiscent of phase-amplitude coupling (right). **(B)** Participants who show a strong response at 40Hz show weaker responses at 60Hz and vice versa. Dots indicate individual participants. Lines reflect the line-of-best-fit computed using least-squares regression.

**Figure S9. Topographic similarities in neural responses to alpha-band stimulation.** To determine whether harmonic responses to stimulation encapsulate effects for one alpha or many, we correlated topographies of fundamental and second harmonic power following stimulation. A raw correlation (left) revealed substantial overlap in all conditions as visual stimulation, regardless of frequency, produced stronger effects in posterior regions relative to frontal regions. Therefore, to remove this confound, we estimated the topographic posterior-to-frontal gradient by averaging across all conditions and then regressed this out on a condition-by-condition basis, using the resulting residuals for topographic correlations (right). to isolate finer-grained topographic structure. After this correction, the harmonic responses at 8Hz, 12Hz, and 16Hz showed substantial topographic overlap with one another, tentatively suggesting that these harmonic responses reflect modulation of a common set of neural generators across conditions. We also observed significant correlations between some fundamental and harmonic topographies (e.g., the 6Hz fundamental with the 16Hz and 20Hz harmonics; the 8Hz fundamental with the 16Hz harmonic), indicating partial spatial overlap between fundamental and harmonic responses. This is consistent with our LDA analyses, which demonstrate that fundamental and harmonic topographies are statistically separable but not entirely non-overlapping. *p_FDR_ < 0.05; **p_FDR_ < 0.01, ***p_FDR_ < 0.001.

**Figure S10. Peak-locking simulation.** Low frequencies dominate neural recordings. This makes it difficult to isolate high-frequency responses without resorting to narrowband filtering. Filtering itself, however, can introduce non-sinusoidal rhythmic artifacts, confounding one of the central questions of this manuscript. To circumvent this issue, we use peak-locking: an iterative method that averages the signal across every peak in the data. Some, however, may be concerned that this introduces artificial rhythmicity (that is, rhythmicity where there was none in the original data). To rule out this possibility, we simulated pink noise (far left column) and gamma bursts (middle left column), then summed them together (middle right column). The ratio of the amplitude of gamma bursts relative to the pink noise was varied from 0 (i.e., no rhythmic activity) to 1 (i.e., substantial rhythmic gamma activity). When peak-locking the data (far right column), we found no rhythmicity in the resulting peak-lock average when there was no rhythmic activity (i.e., top row) nor when rhythmicity was very weak (10% of pink noise; second row). This demonstrates that peak-locked averages are not inherently rhythmic and therefore cannot be seen to introduce artificial rhythmicity.
